# Stromal-induced epithelial-mesenchymal transition induces targetable drug resistance in acute lymphoblastic leukemia

**DOI:** 10.1016/j.celrep.2023.112804

**Published:** 2023-07-14

**Authors:** Chun Shik Park, Hiroki Yoshihara, Qingsong Gao, Chunxu Qu, Ilaria Iacobucci, Pankaj S. Ghate, Jon P. Connelly, Shondra M. Pruett-Miller, Ben Wagner, Camenzind G. Robinson, Ashutosh Mishra, Junmin Peng, Lei Yang, Zoran Rankovic, David Finkelstein, Selina Luger, Mark Litzow, Elisabeth M. Paietta, Nikhil Hebbar, M. Paulina Velasquez, Charles G. Mullighan

**Affiliations:** 1 Department of Pathology, St. Jude Children’s Research Hospital, Memphis, TN 38105, USA; 2 Center for Advanced Genome Engineering, St. Jude Children’s Research Hospital, Memphis, TN 38105, USA; 3 Department of Cell and Molecular Biology, St. Jude Children’s Research Hospital, Memphis, TN 38105, USA; 4 Cell and Tissue Imaging Center, St. Jude Children’s Research Hospital, Memphis, TN 38105, USA; 5 Center for Proteomics and Metabolomics, St. Jude Children’s Research Hospital, Memphis, TN 38105, USA; 6 Department of Structural Biology, St. Jude Children’s Research Hospital, Memphis, TN 38105, USA; 7 Department of Developmental Neurobiology, St. Jude Children’s Research Hospital, Memphis, TN 38105, USA; 8 Department of Chemical Biology and Therapeutics, St. Jude Children’s Research Hospital, Memphis, TN 38105, USA; 9 Department of Computational Biology, St. Jude Children’s Research Hospital, Memphis, TN 38105, USA; 10 Abramson Cancer Center, University of Pennsylvania, Philadelphia, PA 19106, USA; 11 Division of Hematology, Department of Internal Medicine, Mayo Clinic, Rochester, MN 55905, USA; 12 Department of Oncology, Montefiore Medical Center, Bronx, NY 10460, USA; 13 Department of Bone Marrow Transplantation and Cellular Therapy, St. Jude Children’s Research Hospital, Memphis, TN 38105, USA; 14 Lead contact

## Abstract

The bone marrow microenvironment (BME) drives drug resistance in acute lymphoblastic leukemia (ALL) through leukemic cell interactions with bone marrow (BM) niches, but the underlying mechanisms remain unclear. Here, we show that the interaction between ALL and mesenchymal stem cells (MSCs) through integrin β1 induces an epithelial-mesenchymal transition (EMT)-like program in MSC-adherent ALL cells, resulting in drug resistance and enhanced survival. Moreover, single-cell RNA sequencing analysis of ALL-MSC co-culture identifies a hybrid cluster of MSC-adherent ALL cells expressing both B-ALL and MSC signature genes, orchestrated by a WNT/β-catenin-mediated EMT-like program. Blockade of interaction between β-catenin and CREB binding protein impairs the survival and drug resistance of MSC-adherent ALL cells *in vitro* and results in a reduction in leukemic burden *in vivo*. Targeting of this WNT/β-catenin-mediated EMT-like program is a potential therapeutic approach to overcome cell extrinsically acquired drug resistance in ALL.

## In brief

Acute lymphoblastic leukemia (ALL) cell interaction with bone marrow stroma results in drug resistance. Park et al. show that ALL interaction with mesenchymal stromal cells is mediated by integrin beta 1 and drives an EMT phenotype that activates WNT/β-catenin signaling that may be targeted to ameliorate drug resistance.

## Graphical Abstract

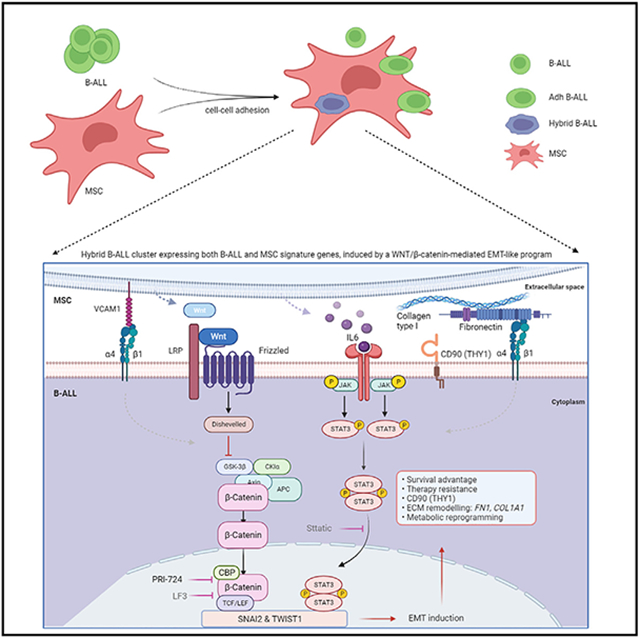

## INTRODUCTION

Acute lymphoblastic leukemia (ALL) comprises multiple subtypes in which a variety of genetic variations have biological and clinical relevance.^1^ B-ALL comprises 23 subtypes defined by genomic alterations that perturb various cellular processes, several of which drive not only leukemogenesis but also cause therapy failure and relapse.^1-3^ Despite advances in ALL treatment, patients with relapsed or refractory ALL still have poor outcomes partly due to cell-intrinsic genetic changes. In addition, prior data suggest that cell-extrinsic cues from the bone marrow microenvironment (BME) contribute to ALL cell survival and chemoresistance.^4,5^ Single-cell RNA sequencing (scRNA-seq) of matched diagnosis and relapsed B-ALL bone marrow (BM) samples has identified remodeling of the myeloid compartment upon disease initiation and subsequent relapse,^6^ but it has not directly interrogated crosstalk between B-ALL cells and non-leukemic cells.

The BME is a complex milieu composed of stromal cells, immune cells, adipocytes, and neural cells^7,8^ that contains niches that modulate leukemia initiation and progression.^9-13^ The BME mesenchymal stem cells (MSCs) and vascular niche cells promote ALL cell survival, quiescence, and therapeutic resistance via soluble factors and adhesion-dependent signaling.^8,12,14-22^ Conversely, leukemic cells reprogram the BME through crosstalk with stromal cells, creating a favorable environment for leukemic cell survival.^23^

We previously showed that cell-intrinsic and -extrinsic pathways intersect to promote resistance in B-ALL. *IKZF1* alterations in high-risk B-ALL result in derepression of stemness and adhesion programs, resulting in BM niche mislocalization, aberrant MSC adherence, and drug resistance.^19^ Limited RNA-seq studies of B-ALL cells revealed chemotherapy-refractory minimal residual disease-like transcriptional signatures in cells adherent to human osteoblast or BM stromal cells.^15^ Additionally, human induced pluripotent stem cell-derived MSCs can protect leukemic cells, both dormant and cycling, against dexamethasone.^24^ Thus, ALL-MSC interactions may promote the survival of ALL cells regardless of genetic subtype, but the molecular mechanisms underlying MSC-induced drug resistance have yet to be elucidated.

Here, we used an *ex vivo* co-culture model to culture primary B-ALL patient samples and cell lines with primary MSCs. Through CRISPR-Cas9 genomic perturbation screens and RNA-seq, we discovered that ALL-MSC interaction induces EMT in ALL cells by binding to MSCs through integrin β1. Additionally, scRNA-seq analyses identified a hybrid cluster of MSC-adherent ALL cells displaying both ALL and MSC gene signatures, induced by EMT activation, with increased β-catenin-mediated transcriptional activity. Inhibiting β-catenin-CBP interaction reduced the survival of patient-derived xenograft (PDX) cells co-cultured with MSCs *ex vivo* and decreased the leukemic burden *in vivo*. These findings highlight the role of ALL-MSC interaction in promoting EMT in ALL cells through WNT/β-catenin signaling, leading to targetable drug resistance.

## RESULTS

### Dynamic change of cell adhesion and extracellular matrix genes in adherent ALL cells

**Figure 1.**
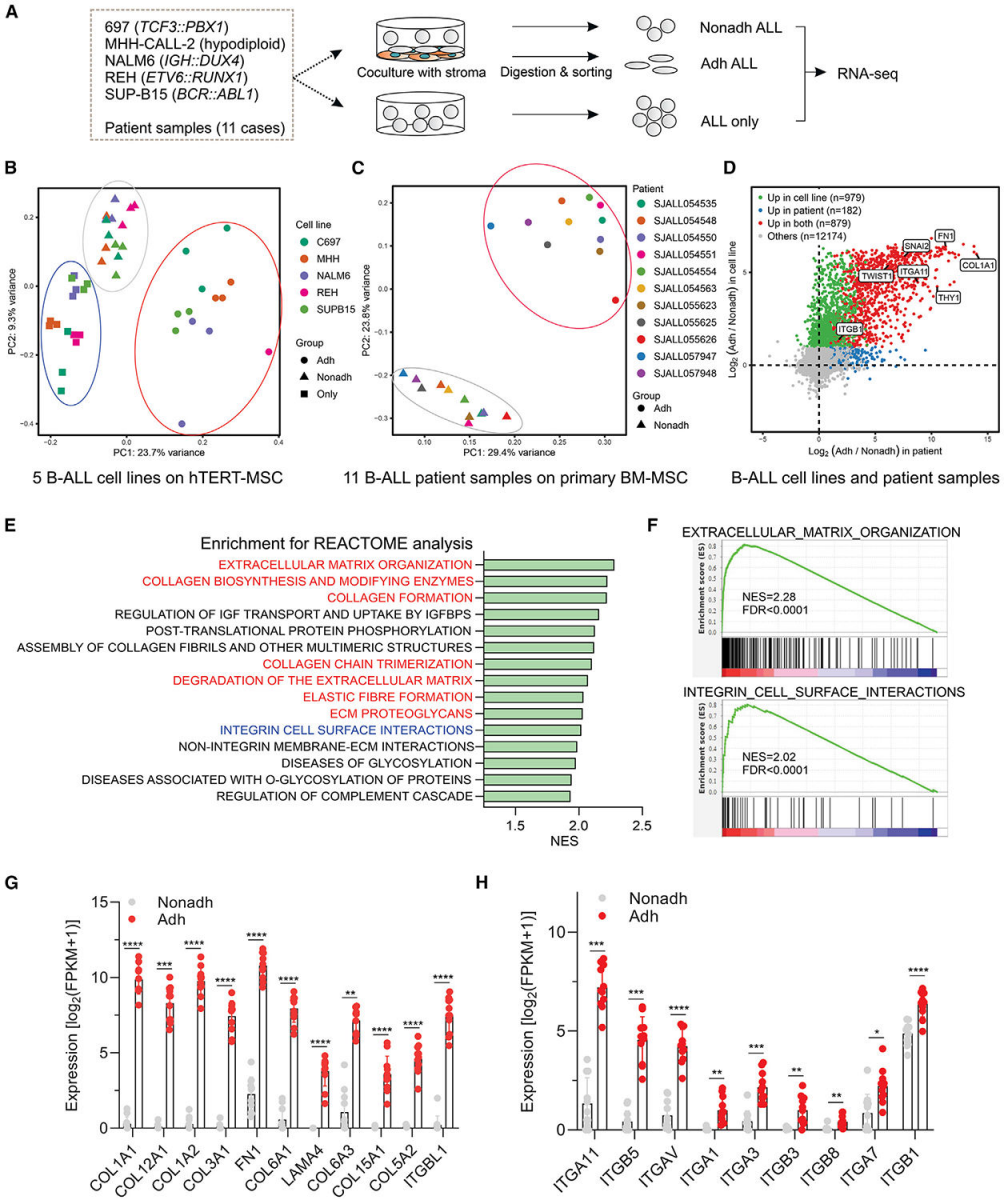
Enrichment of genes encoding cell adhesion molecules and extracellular matrix proteins in adherent ALL cells (A) Schema of *ex vivo* co-culture of ALL with MSCs. Five B-ALL cell lines and 11 B-ALL patient samples were co-cultured with hTERT-MSCs and primary MSCs, respectively. The RNA isolated from ALL-only, Adh ALL, and Nonadh ALL cells was subjected to RNA-seq. (B) PCA plot for five ALL cell lines. (C) PCA plot for 11 primary B-ALLs. (D) Common DEGs in both cell lines and primary patient samples. (E) Pathway analysis of the upregulated DEGs in Adh ALL was performed by using the Reactome database. NES, normalized enrichment score. Red color, ECM-associated pathways. Blue color, integrin-associated pathway. (F) Enrichment plots of integrin cell surface and reactome extracellular matrix organization pathways. FDR, false discovery rate. (G) Enriched integrin family genes in 11 patient Adh ALL samples compared to Nonadh ALL samples (false discovery rate [FDR] < 0.05). Dots, individual value. Bar, mean with SD. (H) Enriched ECM genes in 11 patient Adh ALL samples compared to Nonadh ALL samples (FDR < 0.05). Dots, individual value. Bar, mean with SD. Statistical testing was performed using two-tailed Student’s t test, ****p < 0.0001, ***p < 0.0005, **p < 0.005, *p < 0.05. See also Figure S1.

MSCs are known to contribute to therapy resistance in ALL through direct interactions, the secretion of soluble factors, and formation of tunneling nanotubes.^7,14,25,26^ To investigate MSC-mediated mechanisms of enhanced survival and drug resistance in ALL, we used an *ex vivo* co-culture model of B-ALL cells with BM-derived stromal/stem cells. First, we utilized human telomerase reverse transcriptase-immortalized BM MSCs (hTERT-MSCs)^27^ co-cultured with five B-ALL cell lines of differing genotypes (Figure 1A). ALL cells could be divided into three groups: (1) ALL cells cultured with MSCs that remained nonadherent (Nonadh ALL), (2) ALL cells adherent to MSCs (Adh ALL), and (3) ALL cells cultured without MSCs (ALL only). Nonadh cells in suspension were collected from the co-culture media of ALL with stroma, followed by collection of Adh ALL cells by digestion and cell sorting to >99% purity to avoid hTERT-MSC contamination. Importantly, all ALL lines tested displayed substantial adhesion ability regardless of the type of culture medium, although the REH (*ETV6::RUNX1*) cell line showed less adherence in RPMI than in α-MEM (Figure S1A). Principal component analysis (PCA) of RNA-seq data showed that Adh ALL cells were distinct from both Nonadh ALL and ALL-only cells, which were closely grouped irrespective of leukemia subtype (Figure 1B). There were 2,138 differentially expressed genes (DEGs) between Adh vs. Nonadh ALL cells (2,107 upregulated and 31 downregulated; log_2_(Adh/Nonadh) > 1; FDR < 0.05; Table S1), indicating that ALL-MSC crosstalk primarily augmented ALL gene expression in a subtype-independent manner.

hTERT-MSCs have limitations, including lack of CD90 (THY1)^28-31^ expression, which may bias ALL-MSC interactions. Accordingly, we generated primary MSCs from BM aspirates,^32^ which fulfilled criteria for MSCs,^33^ including adherence, expression of MSC cell surface markers, and the ability to differentiate into osteoblasts, adipocytes, and chondroblasts *in vitro* (Figures S1B-S1D); hTERT-MSCs lacked expression of CD90 (Figure S1E).

These cells were co-cultured with fresh, flow-sorted primary samples obtained at diagnosis from 11 patients with various subtypes of B-ALL (Table S2). In contrast to leukemic cell lines, primary leukemic cells did not grow *ex vivo* without MSCs (Figure S1F) but proliferated over a 4-day period in the presence of MSCs. On gene expression analysis, the 11 Adh ALL samples were grouped together distinct from Nonadh ALL samples (Figure 1C), supporting the notion of subtype-independent deregulation of gene expression driven by ALL-MSC interaction in leukemic cells regardless of the presence of subtype-defining genomic drivers. Differential gene expression analysis identified 1,329 upregulated and 10 downregulated genes in Adh versus Nonadh samples (Table S2), with 879 genes being upregulated in both patient sample and cell lines (Figure 1D). Integrin cell surface interaction and extracellular matrix (ECM) organization pathways were significantly enriched^34^ in Adh cells (Figures 1E and 1F), with upregulation of genes encoding collagen, fibronectin, and laminin and their corresponding ligands including integrin α11, αv, α3, β5, β3, and β1 (Figures 1G and 1H).

### Multiple mechanisms of interaction between ALL and MSCs

**Figure 2.**
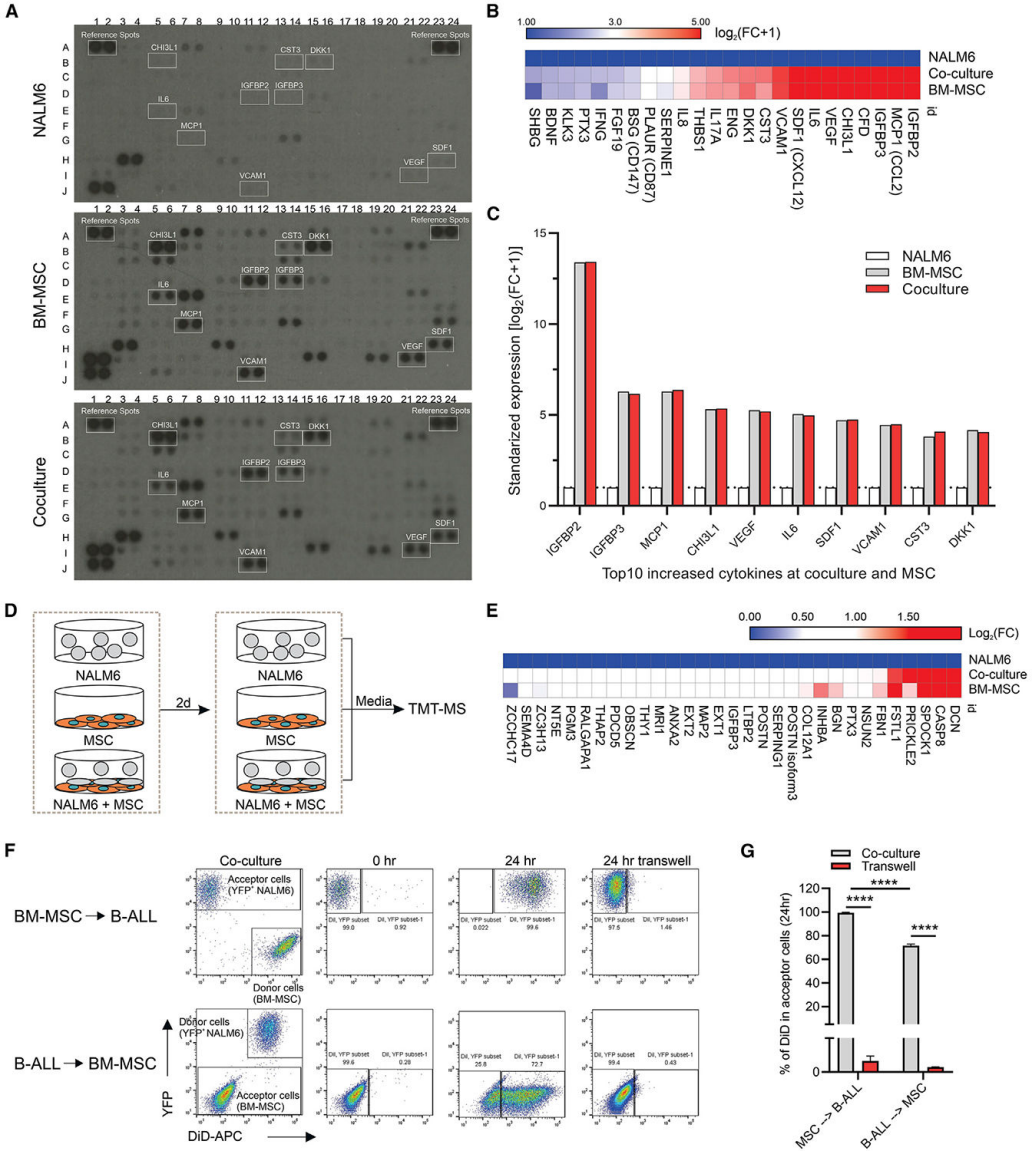
Multiple interactions between B-ALL cells and primary MSCs (A) Identification of enriched cytokines in the co-culture media. (B) All enriched cytokines in the co-culture media compared to the NALM6 media (fold change [FC] > 2.0). (C) Top 10 increased cytokines in the co-culture media. Representative data are from one of two experiments. (D) Scheme of TMT-MS analysis of proteins in the media of NALM6, MSCs, and co-culture of NALM6 and MSCs. (E) Enriched proteins in the co-culture media compared to NALM6 media (FC > 1.5). (F) Dot plots displaying the gating strategy for flow cytometry experiments on TNT-mediated transfer of cellular vesicles. The transfer of the lipophilic membrane stain Dil from labeled donor cells to unlabeled recipient cells was analyzed by flow cytometry. (G) Cumulative efficiency of cellular vesicle trafficking between B-ALL and MSCs (n = 3). Data, mean ± SD. Statistical testing was performed using two-tailed Student’s t test; ****p < 0.0001.

Cytokine array analysis using cytokine-directed antibodies (Table S3) and the NALM6 (*IGH::DUX4*) cell line, which is derived from ALL in relapse,^35^ revealed that 24 cytokines including IGFBP2, VEGF, IL6, and CXCL12 were enriched in MSC-ALL co-culture media compared to NALM6 mono-culture; conversely, no cytokines were enriched in co-culture media compared to MSC mono-culture, indicating that enriched cytokines in co-culture were secreted by MSCs (Figures 2A-2C).

Proteome profiling of media from both mono-culture and co-culture conditions using tandem mass tag mass spectrometry (TMT-MS) (Figure 2D) identified that 31 proteins, including DCN, CASP8, and POSTN (periostin), were enriched in the co-culture media compared to NALM6 mono-culture media (Figure 2E, Table S4). Notably, POSTN derived from MSCs is known to promote B-ALL proliferation by activating the integrin-ILK-NF-κB-CCL2 pathway.^17^ No proteins were enriched by more than 1.5-fold in co-culture media compared to MSC mono-culture (Figure 2E, Table S4) showing that soluble mediators driving ALL-MSC interactions are MSC derived.

Tunneling nanotubes (TNTs), long membrane nanotubes that connect distant cells, have been reported to mediate B-ALL-stroma interaction and drug resistance.^26^ Thus, we evaluated TNT-mediated communication between NALM6 and MSCs and observed bidirectional TNT-mediated B-ALL-MSC interaction (Figures 2F and 2G), in contrast to prior reports of predominantly ALL to hTERT-MSC transfer. Previous studies indicated that TNT signaling between B-ALL and hTERT-MSCs induced prednisolone resistance.^26^ Therefore, we did not test the impact of TNT-mediated B-ALL-MSC interaction on chemoresistance.

### Integrin beta 1 mediates ALL cell adhesion to MSCs

**Figure 3.**
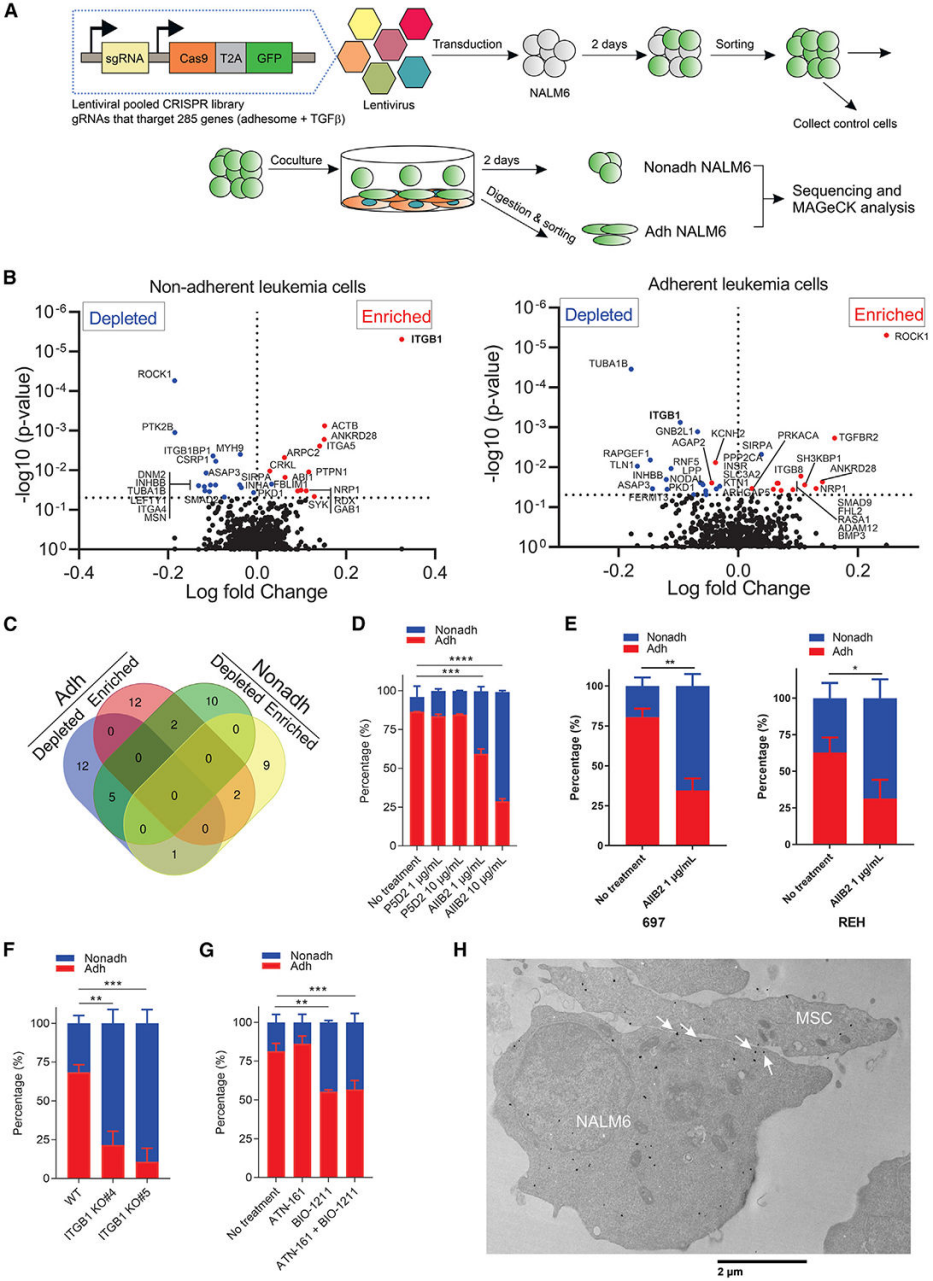
Identification of integrin beta 1 in ALL cells mediating the adhesion to MSCs (A) Functional CRISPR-Cas9 library screening of cell adhesion genes implicated in adhesion of ALL cells to MSCs. NALM6 cells transduced with lentivirus expressing Cas9 and gRNAs that target 285 genes, adhesome (232 genes) and TGFβ pathway (53 genes), were co-cultured with MSCs, and then Nonadh and Adh NALM6 cells were collected for sequencing and MAGeCK analysis. (B) Volcano plots displaying the log fold change on the X axis and −log_10_ p value in Y axis for all sgRNAs identified in Nonadh NALM6 (left panel) and Adh NALM6 (right panel). (C) Venn diagram showing number of genes that are either depleted or enriched genes in both Nonadh and Adh NALM6. (D) Cell adhesion assay of NALM6 cells to MSCs in the presence of P5D2 or AIIB2 (n = 3). Data, mean ± SD. (E) Cell adhesion assay of 697 and REH to MSCs in the presence of AIIB2 (n = 3). Data, mean ± SD. (F) Cell adhesion assay of ITGB1 KO NALM6 clones to MSCs (n = 3). Data, mean ± SD. (G) Cell adhesion assay of NALM6 cells to MSCs in the presence of blocker of either 25 μM ATN-161 or BIO-1211 (n = 3). Data, mean ± SD. (H) TEM of integrin β1 in NALM6 co-cultured with MSCs. White arrows, integrin β1 localized at the synapse between NALM6 and MSCs. Scale bars represent 2 μm. Student’s t test compared to WT or no treatment of Adh leukemic cells, ****p < 0.0001, ***p < 0.0005, **p < 0.005, *p < 0.05. See also Figure S2.

The adhesome is a network comprised of interactions between cellular receptors and components, and TGFβ is known to regulate cell-matrix and cell-cell adhesions.^36^ Thus, to systematically identify cell adhesion genes implicated in adhesion of ALL to MSCs, we performed a CRISPR-Cas9 screen targeting adhesome^37,38^ and TGFβ pathway genes^39^ (Table S5) (Figures 3A, S2A, and S2B). Integrin β1 was the only gene enriched in Nonadh leukemia cell lines and depleted in Adh leukemia cell lines (Figures 3B, 3C, and S2A-S2C). Next, we performed cell adhesion assays in the presence of the integrin β1-blocking antibodies AIIB2 and P5D2 and found that only AIIB2 diminished NALM6, 697, and REH cell adhesion to MSCs in a dose-dependent manner (Figures 3D and 3E).

Moreover, AIIB2 efficiently abrogated NALM6 adhesion onto fibronectin but not P5D2 (Figure S2D). Consistent with these data, AIIB2 reduced adhesion of PDX B-ALL cells to MSCs (Figure S2E). Moreover, integrin β1 knockout (ITGB1 KO) cells (Figure S2F) exhibited strikingly reduced adhesion to MSCs (Figure 3F). We also found that fibronectin blocking peptide (FN1 CS1) and VCAM-1 blocking antibody reduced NALM6 cell adhesion to MSCs (Figures S2G and S2H), indicating that leukemic cells bind to both FN1 and VCAM-1 on MSCs. Integrin α4β1 and α5β1 complexes have been reported to mediate interaction of B-ALL cells to BM stromal cells or chemoresistance.^40-44^ Thus, we performed cell adhesion assays in the presence of the α5β1 blocker (ATN-161) or the α4β1 blocker (BIO-1211).^45^ We found that integrin α4β1 (VLA-4) participated in leukemic cell adhesion to MSCs (Figure 3G). To verify whether integrin α4 (ITGA4) is implicated in the adhesion of leukemia cells to MSCs, we knocked down ITGA4 and ITGA5 and examined cell adhesion. Despite downregulation of protein level (Figure S2I), knockdown had only a modest effect on cell adhesion (Figure S2J). In contrast, ITGA4 knockdown augmented ITGA5 expression, indicating that ITGA4 knockdown results in compensatory deregulated expression of other integrins (Figure S2K). Using an ECM array slide, NALM6 exhibited strong affinity to fibronectin and fibronectin-included ECM combinations and weak adhesion to collagen I-included ECM combinations (Figure S2L). Using immunofluorescence, we observed that integrin β1 protein was redistributed from diffuse cytoplasmic location to contacts between NALM6 and MSCs upon engagement with MSCs (Figure S2M). Further, transmission electron microscopy (TEM) analysis showed that integrin β1 was located in the synapse between NALM6 and MSCs (Figure 3H). Collectively, these data demonstrate that ALL cells adhered to MSCs via integrin β1 localized at the synapse between ALL cells and MSCs by binding to fibronectin and VCAM-1 on MSCs.

Next, we tested whether integrin β1-mediated cell adhesion is implicated in the chemoresistance of NALM6 cells co-cultured with MSCs using ITGB1 KO NALM6 cells expressing yellow fluorescent protein (YFP) and firefly luciferase (ffLuc). ITGB1 KO did not sensitize NALM6 cells co-cultured with MSCs to dexamethasone (DEX) or cytarabine (Figures S3A and S3B). However, integrin β1 is also critical for multiple various cell functions, including survival, homeostasis, and cell adhesion,^46^ and we observed poor cell viability of ITGB1 KO NALM6 cells even in the absence of drugs and MSCs (Figures S3A and 3B). Therefore, we also performed cell viability assays in the presence of blockers of integrin β1-mediated adhesion. Both the blocking antibody of integrin β1 (AIIB2) and the inhibitor of integrin α4β1 (BIO-1211) sensitized YFP-positive NALM6 cells co-cultured with MSCs (Figures S3C and S3D), indicating that the cell adhesion of ALL cells to MSCs via integrin β1 as part of the α4β1 complex contributes to the chemoresistance of B-ALL cells in contact with MSCs.

### ALL-MSC interactions drive an EMT-like program in ALL

**Figure 4.**
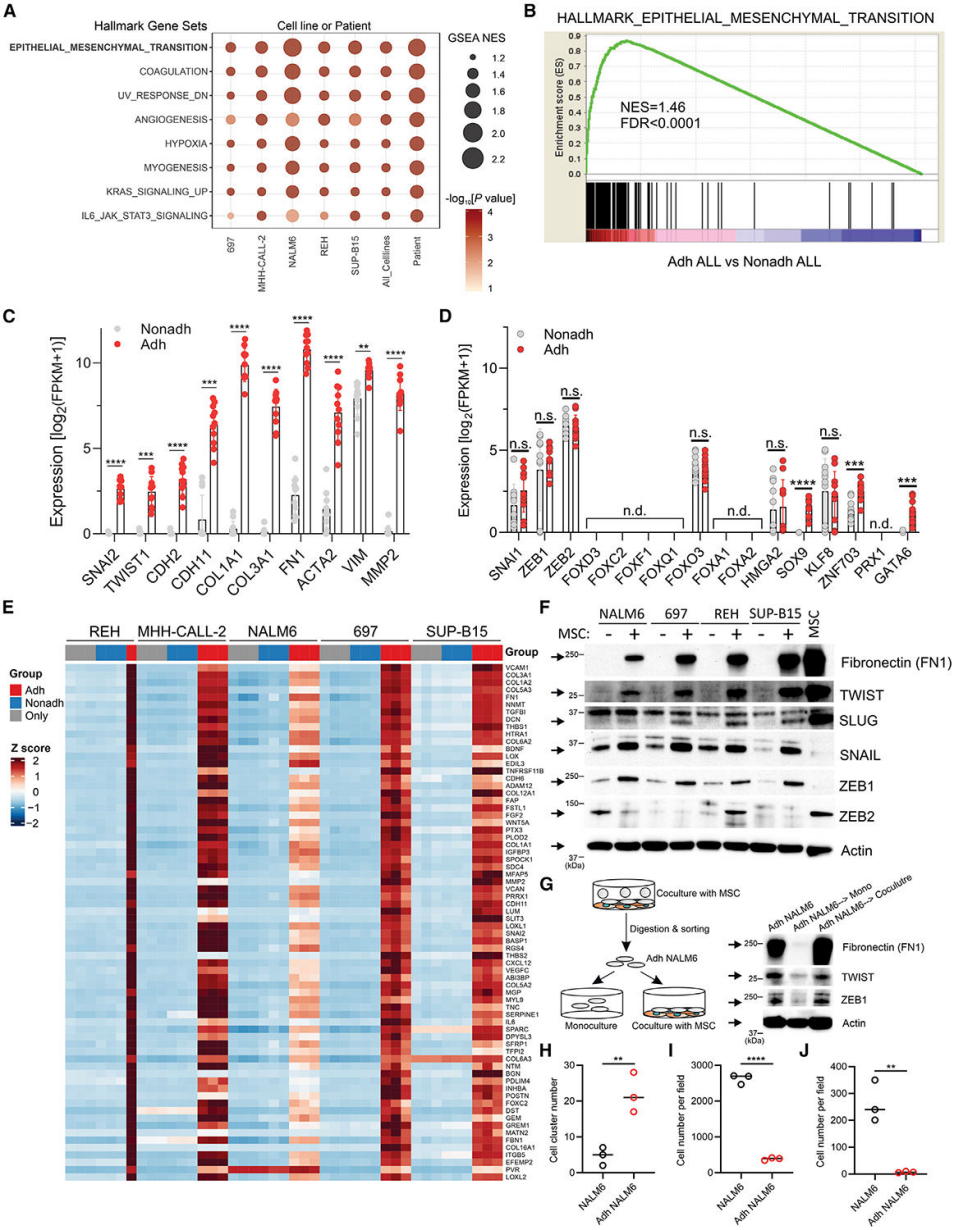
Induction of an EMT-like program in Adh ALL cells (A) Gene set enrichment analysis against hallmark gene sets, comparing Adh ALL with Nonadh ALL, for each cell line, combined all cell lines as well as combined 11 patients. Only gene sets with FDR < 0.05 in at least one comparison and nominal p value < 0.01 in at least four comparisons were selected. (B) Enrichment plot of hallmark EMT dataset enriched in Adh ALL cell lines. (C) The upregulation of EMT markers, including SNAI2, TWIST1, CDH2, FN1, and MMP2, was determined by mRNA-seq in 11 patients with Adh ALL (FDR < 0.05). Data, mean ± SD. (D) Expression level of conventional and novel EMT TFs in 11 patient Adh ALL samples. n.d., not detectable. n.s., not significant. Data, mean ± SD. (E) Heatmap of top 70 gene set enrichment analysis EMT genes in five Adh ALL cell lines. (F) Immunoblotting analysis of EMT markers in Adh cells. (G) Analysis of the EMT state in mono- or co-cultured YFP^+^ NALM6 cells with MSCs for 48 h by immunoblotting. (H) Cell aggregation assay of Adh YFP^+^ NALM6 cells (n = 3). Y axis shows the number of cell clusters consisting of more than four cells. Data, mean with individual value. (I and J) Cell migration and invasion assay of Adh YFP^+^ NALM6 cells, respectively (n = 3). Y axis shows the number of YFP^+^ NALM6 cells that migrated from the upper chamber and subsequently bound to MSCs. Data, mean with individual value. Statistical testing was performed using two-tailed Student’s t test, ****p < 0.0001, ***p < 0.0005, **p < 0.005. See also Figures S4A-S4D.

Pathway analysis of genes deregulated upon ALL-MSC engagement of five ALL cell lines and 11 patient samples showed EMT to be the top enriched gene set (NES = 1.46; FDR < 0.0001) in Adh ALL samples (Figure 4A and 4B). Enriched genes in Adh ALL patient samples included the EMT transcription factors (TFs) *SNAI2* and *TWIST1*, the cadherins *CDH2* and *CDH11*, collagens *COL1A1* and *COL3A1*, *FN1* (fibronectin), *ACTA2* (actin), and *MMP2* genes (Figure 4C). Additional TFs known to activate EMT^47^ including *SOX9, ZNF703*, and *GATA6* were also overexpressed in Adh patient samples (Figure 4D). This striking upregulation of EMT genes in Adh ALL cells is shown in a gene expression heatmap of five ALL cell lines (Figure 4E).

Immunoblotting showed the EMT makers fibronectin (*FN1*), TWIST (*TWIST1*), and SLUG (*SNAI2*) were highly expressed in the five Adh ALL cell lines (Figure 4F). Although SNAI1, ZEB1, and ZEB2 were not upregulated in RNA-seq data of Adh ALL patient samples (Figure 4D), they have putative roles in leukemia development/progression,^48-50^ and we observed SNAIL (*SNAI1*) and ZEB1 but not ZEB2 to be highly expressed in Adh leukemic cell lines (Figure 4F). Interestingly, SNAIL and ZEB1 proteins were not highly expressed in MSCs, in contrast to TWIST and SLUG. We also tested if the EMT property of Adh ALL cells was reversible by measuring the EMT markers in Adh leukemic cells after additional culture with or without MSCs. The protein levels of the EMT markers in Adh ALL cells were diminished after mono-culture for 48 h, while they were maintained in Adh ALL cells after co-culture with MSCs, indicating that MSC-derived EMT in Adh leukemic cells is dynamic and reversible (Figure 4G). Next, we found that Adh ALL cells form larger clusters compared to mono-cultured ALL cells (Figures 4H and S4A). Consistent with this, a substantial EMT phenotype of Adh ALL cells was retained after mono-culture for 24 h, with a decline after 48 h (Figures 4G and S4B). Surprisingly, the migration and invasion ability were suppressed in Adh ALL cells (Figures 4I, 4J, S4C, and S4D). Collectively, this indicates that elevated adherence in Adh ALL cells promotes aggregation, which in turn impedes migration and invasion. As ALL-MSC interactions drive an EMT-like state, which is strongly associated with the maintenance of stemness, modulation of metabolism, and drug resistance,^51^ we hypothesized that MSC-induced EMT in B-ALL might be implicated in the survival and drug resistance of B-ALL.

### Hybrid ALL-MSC-adherent cells driven by an EMT-like program

**Figure 5.**
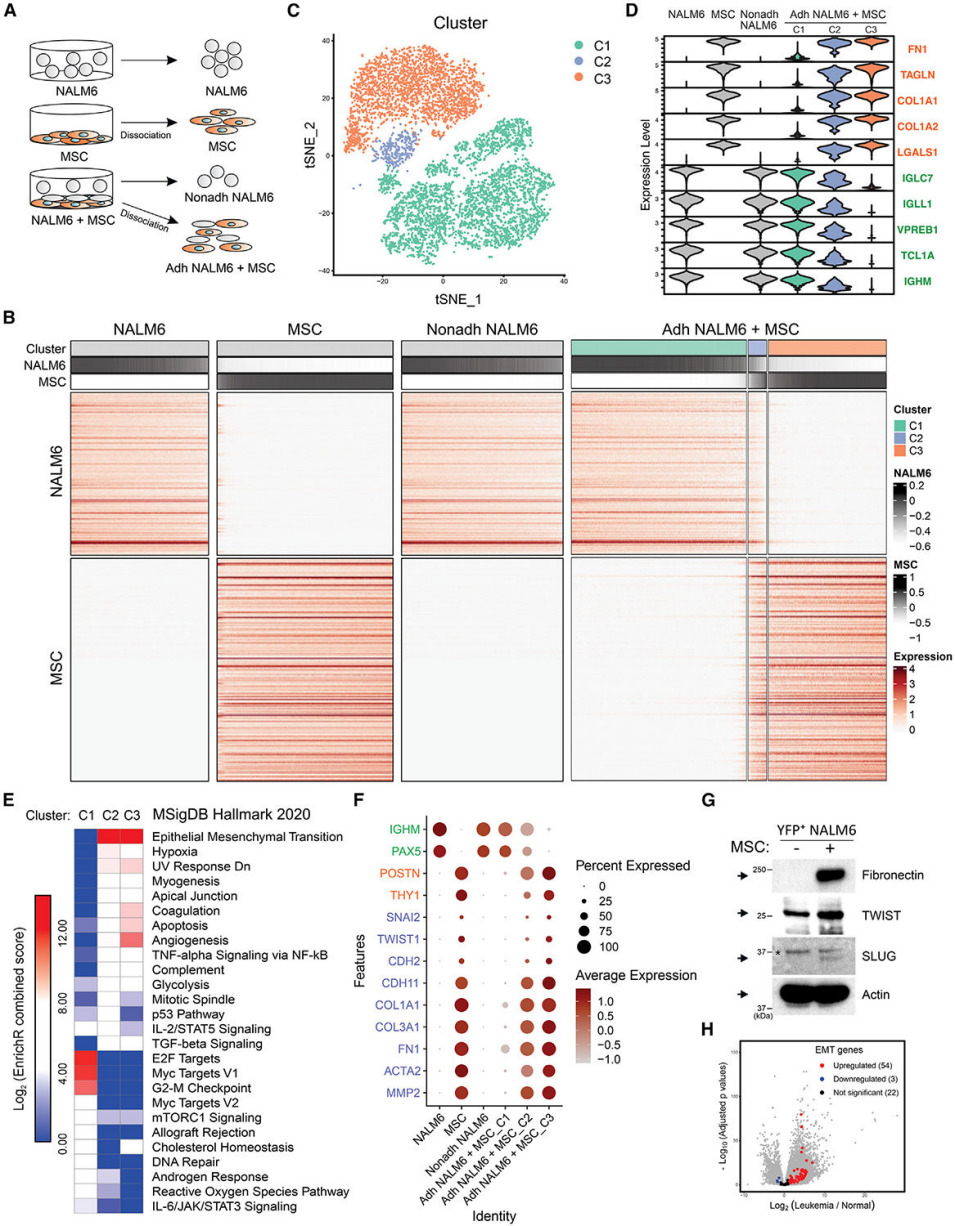
Identification of novel hybrid cluster retaining both ALL and MSC features in Adh ALL cell-driven EMT-like program by scRNA-seq analysis (A) Scheme of droplet-based scRNA-seq analysis of NALM6, MSCs, Nonadh NALM6, and Adh NALM6 plus MSCs. (B) Heatmap showing normalized expression of the NALM6 and MSC signature genes across cells from all four samples. Cell cluster for Adh NAML6 plus MSC sample and aggregated signature scores are shown on the top. (C) tSNE plot showing three clusters of cells in Adh NALM6 plus MSC sample. (D) Violin plot showing expression pattern of top five genes of NALM6 and MSC signature across all the samples/clusters. (E) Enrichment analysis for MSigDB Hallmark 2020 analysis in clusters C1, C2, and C3 of Adh NALM6 plus MSC samples by analyzing top 100 genes of each cluster using EnrichR. Log_2_ (enrichment combined score). (F) Dot plots showing the expression of EMT, MSCs, and B-ALL markers, respectively (Y axis) in each cluster (X axis). The size of the dot represents the percentage of cells expressing the gene, and saturation of color represents average normalized expression level. (G) Immunoblotting analysis of the levels of FN1, TWIST, SLUG, and actin in YFP-positive NALM6 cells with (+) or without (−) MSCs. Asterisk(*), nonspecific bands. (H) Volcano plots displaying the expression level of EMT genes, which were enriched hybrid clusters, is enriched in patient sample compared to normal BM-derived CD19^+^ cells. See also Figures S4E and S4F.

To characterize the heterogeneity of ALL cells induced by interaction with MSC, we performed scRNA-seq of NALM6 cells (1) and MSCs (2), each cultured in isolation, and from co-culture of NALM6 cells with MSCs, Nonadh NALM6 (3), and Adh NALM6 plus MSCs (4) (Figure 5A). We generated a heatmap showing normalized expression of NALM6 and MSC signature genes across cells from all samples (Figure 5B). Consistent with bulk RNA-seq, the Nonadh NALM6 sample displayed a similar expression signature to NALM6 alone. In the NALM6-MSC co-culture, we observed a population of cells expressing both NALM6 and MSC signature genes (cluster 2, C2), in addition to the expected NALM6 and MSC populations, which was also evident in a t-distributed stochastic neighbor embedding (tSNE) plot analysis of scRNA-seq data (Figures 5B and 5C). Cells in this hybrid cluster expressed hallmark B-ALL genes such as *IGLC7, IGLL1, VPREB1, TCL1A*, and *IGHM*, albeit at lower levels than NALM6 (C1), and expressed MSC genes such as *FN1, TAGLN, COL1A1, COL1A2*, and *LGALS1* (Figure 5D), indicating that this hybrid cluster (C2) is derived from B-ALL cells but had acquired MSC features.

Pathway analysis using the top 100 expressed genes in each cluster showed that the hybrid C2 cluster exhibited evidence of EMT signaling that was not present in the remaining NALM6 cells (C1 cluster; Figure 5E). Thus, the EMT observed in Adh ALL elicited by crosstalk with MSCs is heterogeneous and likely mediates acquisition of hybrid phenotype of the C2 cells that exhibit expression of B-ALL and MSC genes. In addition, hypoxia and glycolysis pathways, which contribute to the emergence of stem cell-like leukemia-initiating cells in B-ALL,^52^ were also found to be enriched in the cluster C2 compared to C1 (Figure 5E).

In contrast, cell proliferation-related pathways (E2F targets, MYC targets V1, and G2-M checkpoint genes) were enriched in C1 (Figure 5E). Consistent with induction of EMT, we observed upregulation of the EMT genes *SNAI2, TWIST1, CDH2, CDH11, COL1A1, COL3A1, FN1, ACTA2*, and *MMP2*, in hybrid C2 cells, which were also upregulated in MSC-adherent patient ALL cells in bulk RNA-seq analyses (Figure 4C) but not in C1, NALM6, and Nonadh NALM6 samples (Figure 5F). Collectively, these data indicate EMT activation contributes to the formation of the hybrid C2 cell cluster.

To examine whether the novel hybrid C2 cluster is ALL derived, and to exclude the possibility of contamination by MSC, we measured the protein expression level of fibronectin, TWIST, and SLUG, which were upregulated EMT markers in the hybrid cluster, in YFP-positive NALM6 cells. Indeed, the levels of these proteins were elevated in Adh YFP-positive NALM6 cells (Figure 5G), indicating that the hybrid cluster was derived from ALL via activation of EMT and previous findings were not due to MSC contamination. Consistent with this, most of the MSC-derived genes expressed in the hybrid cluster (Figure 5B) were also deregulated in bulk RNA-seq data from both cell line and patient samples and were components of the EMT, coagulation, and hypoxia hallmark pathways enriched in bulk RNA-seq data (Figure S4E).

The majority of EMT genes (50/70) were also upregulated in patient samples compared to normal BM, emphasizing the clinical relevance of these findings (Figure 5H). To examine whether this hybrid population could be identified by immunophenotyping, we examined ITGAV, a cell surface protein enriched in hybrid cluster (Figure S4E), and found ITGAV to be highly expressed in Adh NALM6 compared to NALM6 alone (Figure S4F).

### Targeting of EMT-associated signaling pathways impairs leukemic cell viability

**Figure 6.**
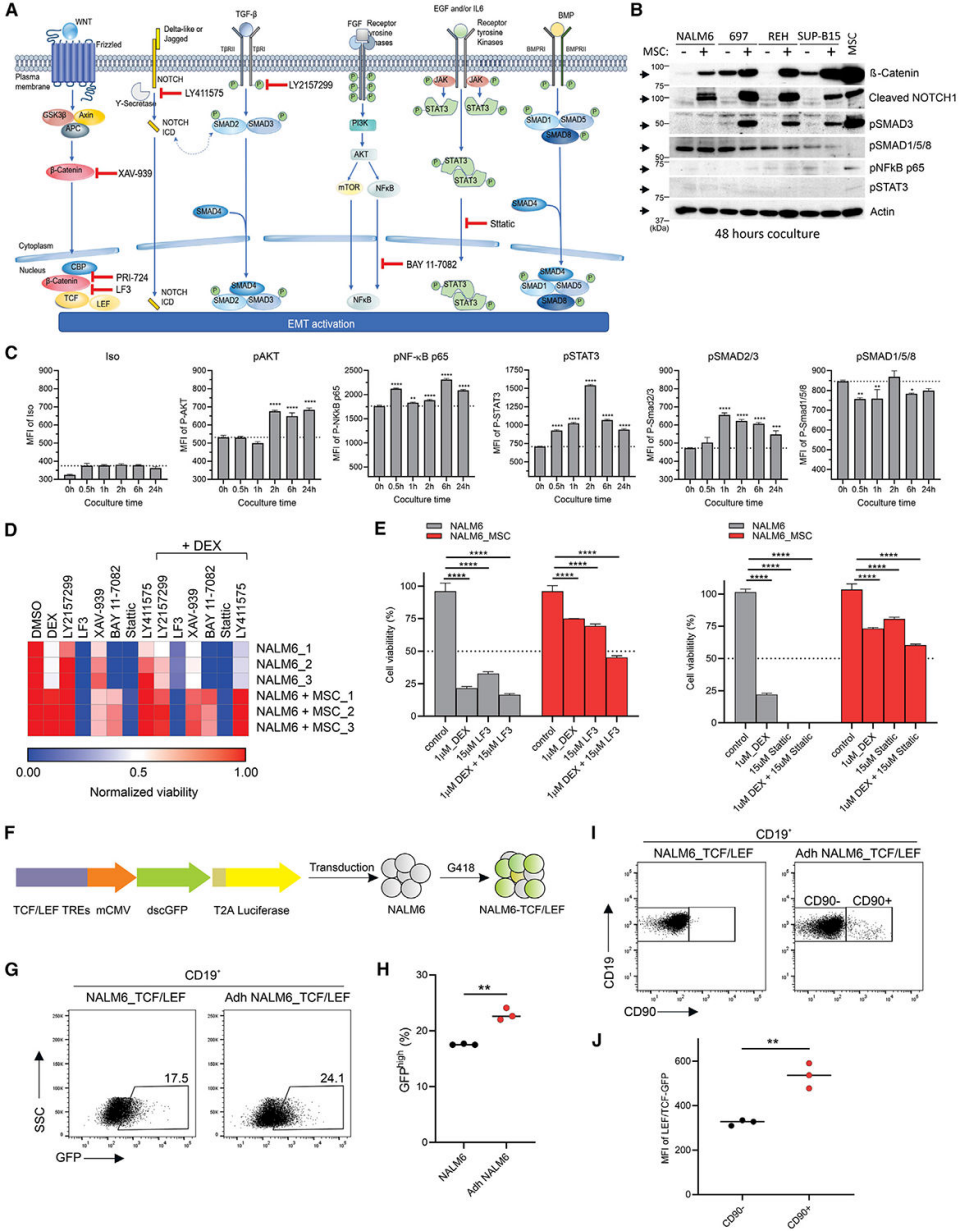
Targeting WNT/β-catenin and STAT3 signaling pathways impairs cell viability of Adh cells (A) Signaling pathways leading to EMT activation based on two review journals.^53,54^ Inhibitors of each signaling pathways are shown. (B) Immunoblotting analysis of the levels of fibronectin, TWIST, SLUG, β-catenin, cleaved NOTCH1, pSMAD3 (pS423/S425), pSMAD1/5/8 (pS463/S465 on SMAD1, pS463/S465 on SMAD5, pS466/S467 on SMAD8), pNF-κB p65 (pS536), pSTAT3 (pY705), and actin in Adh ALL cells after 48 h of co-culture with MSCs. "p," an abbreviation for "phosphorylated." (C) Phospho-flow cytometry in Adh NALM6 at indicated time. All samples were analyzed for the mean fluorescence intensity (MFI) of pAKT (pS473), pNF-κB p65, pSTAT3, pSMAD2/3 (pS465/S467 on SMAD2, pS423/S425 on SMAD3), and pSMAD1/5/8 in Adh NALM6. (n = 3). Data, mean ± SD. Statistical testing was performed using two-tailed Student’s t test; ****p < 0.0001, ***p < 0.0005, and **p < 0.005. (D) Cell viability assay in the presence of inhibitors of activated signaling pathways in Adh cells with or without DEX. DEX = 1 μM and other drugs = 40 μM (E) Effects of LF3 and Stattic in ALL cells with or without DEX (n = 3). Data, mean ± SD. Significance was assessed by two-way ANOVA with a Sidak adjustment for multiple comparison between control and drug-treated samples, ****p < 0.0001. (F) Generation of NALM6-TCF/LEF reporter cell line expressing TCF/LEF transcriptional response elements. (G) Upregulated β-catenin-mediated transcriptional activity in Adh NALM6 compared to NALM6. For analysis, the signal from NALM6, CD19^+^ cells is gated, and then GFP signals were measured. (H) Analysis of β-catenin-mediated transcription activity in Adh NALM6 by measuring percentage of relatively high level of GFP signal (GFP^high^) (n = 3). Data, individual value and mean. (I) Flow cytometry of CD90 in NALM6 and Adh NALM6 after gating CD19^+^. (J) Flow cytometric measurement of β-catenin-mediated transcriptional activity in either CD19^+^CD90− or CD19^+^CD90^+^ subset of Adh NALM6 by assessing MFI of GFP (n = 3). Data, individual value and mean. Statistical testing was performed using two-tailed Student’s t test, **p < 0.005. See also Figures S5 and S6.

Several signaling pathways collaborate to induce EMT in response to signaling cues from the BME, including WNT, NOTCH1, TGFβ, receptor tyrosine kinase (RTK), cytokine signaling, and BMP^53,54^ (Figure 6A). We investigated activation of these pathways in Adh ALL cells by measuring expression of the main executor protein of each pathway: β-catenin (WNT signaling), cleaved NOTCH1 (NOTCH1 signaling), phosphorylated SMAD3 (pSMAD3) (TGFβ signaling), pNF-κB p65 (RTK signaling), pSTAT3 (cytokine signaling), and pSMAD1/5/8 (BMP signaling). Unexpectedly, several signaling pathways known to drive EMT, including WNT, NOTCH1, and TGFβ, were activated in Adh ALL cell lines, based on increase of protein level of β-catenin, cleaved NOTCH1, and pSMAD3, respectively (Figure 6B). We also examined the level of phosphorylation of AKT, NF-κB p65, STAT3, SMAD2/3, and SMAD1/5/8 at earlier time points to detect transient activation. We observed activation of RTK (pAKT and pNF-κB p65), cytokine signaling (pSTAT3), and TGFβ (pSMAD2/3) at earlier time points (Figure 6C). Various cells in the BME secrete IL6, leading to activation of STAT3 signaling in tumor cells^55^; moreover, we observed enrichment of IL6 in co-culture media due to secretion from MSCs (Figures 2B and 2C) that demonstrated that IL6 secreted from MSCs led to transient activation of STAT3 signaling in Adh ALL cells.

As expected, NALM6 co-cultured with MSCs displayed strong resistance to DEX compared to NALM6 (Figure S5A). Next, to determine which of the WNT, NOTCH1, TGFβ, and cytokine signaling pathways activated in Adh ALL mediated leukemic cell survival and drug resistance, we performed cell viability assays in the presence of inhibitors (Figure 6A). To prioritize pathways for detailed investigation, we first used inhibitors of activated signaling pathways at high concentration (40 μM; Figure 6D) and 1 μM DEX. Notably, only inhibitors of WNT (the β-catenin-TCF inhibitor LF3 and the stimulator of β-catenin degradation by stabilization of Axin, XAV-939) and cytokine signaling (the inhibitor of nuclear translocation pSTAT3, Stattic) reduced viability of NALM6 cells co-cultured with MSCs. The inhibitor of nuclear translocation of NF-κB, BAY 11–7082, was only effective in NALM6 mono-culture (Figure 6D), indicating that activated WNT and cytokine signaling pathways represent potential targets to eradicate B-ALL cells adherent to MSCs. Thus, we tested the WNT inhibitor LF3 and cytokine signaling inhibitor Stattic at 15 μM concentration alone or with 1 μM DEX. Both drugs reduced cell viability as single agents and with enhanced toxicity in combination with DEX, with greatest efficacy for the WNT pathway inhibitor LF3 in NALM6-MSC co-culture (Figure 6E).

Cell viability assay using YFP-positive NALM6 cells following TWIST1 or SNAI2 siRNA-mediated knockdown, which sensitized NALM6 cells co-cultured with MSCs to DEX (Figures S5B and S5C), demonstrates that the upregulation of TWIST1 and SNAI2 in Adh ALL cells is implicated in MSC-mediated drug resistance. We investigated whether DEX treatment could potentiate EMT in NALM6 cells that were co-cultured with MSCs by enriching Adh ALL cells with EMT phenotype by treating the cells with either vehicle or 50 nM DEX (the IC_75_ of DEX in NALM6 mono-culture) and found that DEX treatment slightly increased EMT, as evidenced by the increased fibronectin expression in DEX-treated NALM6 with MSCs (Figure S5D).

### MSCs confer dormancy, proliferation, and senescent characteristics to adherent ALL cells

We investigated the cell-cycle status of NALM6 and Adh NALM6 cells, showing that Adh ALL cells had increased dormant (G_0_) and proliferative (S-G_2_/M) populations (Figure S6A), both of which were also increased in MSC-co-cultured ALL PDX cells (Figure S6B). Consistent with these results, there was upregulation of both cell-cycle promoting (CCND1, CDK15, and CDK20) and inhibitory genes (CDKN2B and CDKN2A) in Adh leukemic cell lines (Figure S6C). Thus, Adh leukemic cells were heterogeneously composed of chemoresistant and proliferative subsets. We also found that MSCs protect Adh PDX cells from apoptosis (Figure S6D). The senescence-associated β-galactosidase activity assay showed that senescence program was augmented in Adh PDX cells (Figure S6E). Consistent with these data, many senescence-associated secretory phenotype genes were enriched in Adh cell lines (Figure S6F).

### β-Catenin-mediated transcription activity is enhanced in adherent and hybrid ALL cells

The canonical WNT/β-catenin signaling pathway involves the nuclear translocation of β-catenin and activation of target genes via TCF/LEF TFs in response to WNT ligands.^53^ Accumulation of β-catenin in Adh ALL cells and inhibition of its interaction with TCF reduced cell viability, indicating that increased β-catenin forms a complex with TCF/LEF, leading to transcription activation in Adh ALL cells. Thus, we established a NALM6 reporter cell line (NALM6-TCF/LEF) expressing TCF/LEF transcriptional response elements under control of the minimal CMV (mCMV) promoter and dscGFP-T2A-luciferase, which was used to assess β-catenin-mediated transcriptional activity by measuring GFP or luciferase activity (Figure 6F). We observed increased GFP-positive cells in NALM6-TCF/LEF co-cultured with MSCs (Figures 6G and 6H).

Prior data showed that THY1 (CD90) is transcriptionally repressed by IKZF1 (Ikaros) and is overexpressed in drug-resistant, *IKZF1*-altered *BCR::ABL1* ALL.^19^ As we here observed that THY1 is enriched in the hybrid cluster C2 (Figure 5F), we tested if THY1 could be used as a cell surface marker for this hybrid cluster. THY1 protein expression was increased in Adh ALL cells (Figure 6I), and the CD90-positive subpopulation of Adh NALM6-TCF/LEF cells displayed elevated β-catenin-mediated transcriptional activity (Figures 6I and 6J) demonstrating that the hybrid cluster is dependent upon on WNT/β-catenin-mediated transcriptional activity.

### Inhibition of β-catenin-CBP interaction reduces the viability of B-ALL cells co-cultured with MSCs

**Figure 7.**
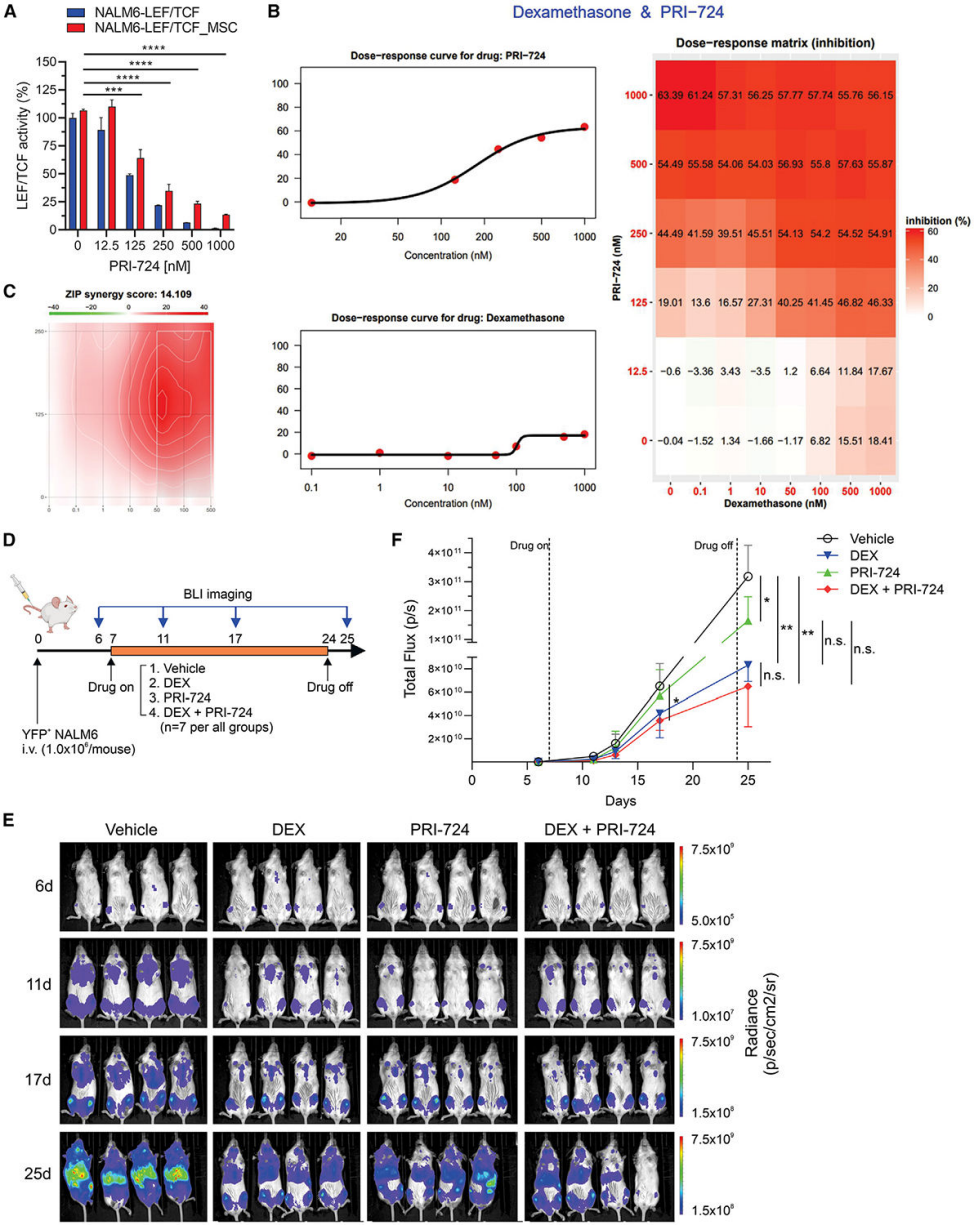
Targeting of β-catenin-CBP interaction reduces leukemic burden of B-ALL mouse model and inhibits the viability of B-ALL PDX cells co-cultured with MSCs (A) Inhibition of β-catenin-mediated transcriptional activity by PRI-724 (n = 3). Data, mean ± SD. Statistical testing was performed using two-tailed Student’s t test; ****p < 0.0001 and ***p < 0.0005. (B) Dose-response matrix (inhibition) for PRI-724 and DEX in YFP-positive NALM6 cells co-cultured with MSCs. The left two graphs show dose-response curves for PRI-724 (upper) and DEX (lower). Y axis shows the inhibition rate. The right graph shows drug response of combination of both drugs. Inhibition scores are analyzed by SynergyFinder 2.0. (C) ZIP synergy score used for synergy score. Less than −10, antagonistic; from −10 to 10, additive; larger than 10, synergistic. (D) Schematic of the NALM6 xenograft model. NSG mice were treated with vehicle, DEX (4 mg/L), PRI-723 (40 mg/kg), or combination of both drugs on day 7 and stopped at day 24. Leukemic burdens were analyzed by BLI. (E) BLI images of day 6, 11, 17, and 25. (n = 7 per group). (F) Quantification of the total flux (p/s) of BLI. Data, mean ± SD. Statistical testing was performed using two-way ANOVA with Tukey’s multiple comparisons test, **p < 0.005, *p < 0.05. See also Figure S7.

Targeting of WNT signaling by inhibition of β-catenin-TCF interaction was effective in reducing viability of MSC-adherent ALL cells but at relatively high inhibitor concentration, so we sought alternative approaches to target this signaling pathway. For example, blocking of β-catenin-CBP interaction reverses pulmonary fibrosis, which is mediated by EMT^56^; and the β-catenin-CBP complex promotes self-renewal of stem cells, while β-catenin-p300 complex induces differentiation.^57^ Moreover, targeting of β-catenin-CBP interaction reduces drug resistance of chronic myeloid leukemia and the survival of leukemic stem cells in acute myeloid leukemia.^58,59^ Therefore, we explored if WNT/β-catenin target genes associated with stemness and self-renewal were enriched in Adh ALL cells.^60^ Expression of *FN1, SNAI2, HAS2, DCLK1, DDND1, CD44, CLDN1, DKK1*, and *TERT* was enriched in all five Adh cell lines (Figure S7A), and expression of genes with promoter TCF4 binding sites was also enriched in Adh ALL cell lines (Figure S7B). PRI-724, a selective inhibitor of β-catenin-CBP interaction, reduced the β-catenin-mediated transcriptional activity of NALM6 co-cultured with MSCs at nanomolar range (IC_50_ = 172 nM; Figure 7A), and in contrast to DEX, it inhibited the viability of NALM6 cells co-cultured with MSCs as a single agent (Figure 7B). In addition, PRI-724 was synergistic with DEX to inhibit the cell viability of Adh NALM6 cells (Figures 7B and 7C).

### PRI-724 reduces the leukemic burden of cell-line-derived xenograft mice

Pharmacokinetic (PK) studies showed that the concentration of C-82, a dephosphorylated form of PRI-724, in the plasma treated with 40 mg/kg of PRI-724 reached 185 nM (Figures S7C and S7D), which might be enough to target Adh NALM6 cells (IC_50_ = 172 nM; Figure 7B). However, it rapidly degraded within 6 h. Despite low stability of PRI-724/C-82 *in vivo*, we tested the efficacy of PRI-724 as a single drug or in combination with DEX using NALM6 xenograft model by measuring leukemia burden with bioluminescence imaging (BLI) (Figure 7D). The data showed that PRI-724 was able to reduce leukemia burden compared to the vehicle control (Figures 7E and 7F). When combined with DEX, there was a trend toward further reduction in tumor burden over 4 weeks of therapy, compared to the single-drug regimen groups. These results suggest that targeting the β-catenin-CBP interaction is effective *in vivo*, but stable drug is required to fully target β-catenin-CBP-mediated drug resistance.

Finally, we assessed the ability of PRI-724, either as a single drug or in combination with DEX, to inhibit the viability of B-ALL PDX leukemic cells co-cultured with MSCs *ex vivo* to faithfully model human leukemic cell-BME interactions. PRI-724 as a single drug displayed antileukemic activity in all B-ALL PDX models (Figures S7E and S7F), with two types of responses in combination with DEX: either more potent activity (Figure 7E) or no greater activity compared to PRI-724 alone (Figure 7F). These results suggest that targeting the β-catenin-CBP interaction is effective in alleviating DEX-resistant Adh ALL cells, either as a single agent or in combination with DEX. The varying efficacy of PRI-724 in combination with DEX among PDX samples may be attributed to specific ALL subtype-asociated genomic alterations.

## DISCUSSION

Prior studies have implicated cellular components of the BME as influencing ALL cell survival and drug responsiveness and have nominated specific mediators of interaction (e.g., integrin-mediated adherence or intercellular tubule formation) and leukemic cell tropism (e.g., provision of amino acids such as asparagine).^5,13,16,26,42,43,61^ However, most prior studies have focused on individual pathways of interaction and have commonly been limited by analysis of cell lines,^6,15^ which may not fully reflect leukemia biology *in vivo* as such lines have lost the requirement for stromal cell interaction to proliferate.

To address these issues, we detailed analyses characterizing the mechanisms of interaction and the alterations in leukemia cell phenotype and resistance using complementary experimental approaches: comparison of leukemia and stromal cell line interactions with those of primary leukemia cell lines and stroma, and genomic analyses at the bulk and single-cell levels, coupled with interrogation and perturbation of implicated downstream signaling pathways. Collectively, the results were striking in the diversity of pathways and mechanisms perturbed at leukemia cell engagement with stroma. These include induction of a gene expression signature primarily in leukemic cells characterized by EMT, coagulation, and hypoxia, several of which, including EMT, are known to mediate tumor resistance and metastasis in other cancer contexts.^51^ scRNA-seq of isolated ALL cell line, MSC, and ALL-MSC co-culture demonstrated that a subset of MSC-adherent leukemia cells adopt hybrid ALL-MSC characteristics, and that EMT is a driver of acquisition of this phenotype. Although only a minority of MSC-adherent leukemia cells in the adherent cell population exhibited this hybrid phenotype on scRNA-seq, these analyses likely underestimate the magnitude of this phenomenon, as we showed it to be dependent on integrin-mediated cell adhesion (which is disrupted on harvesting cells for scRNA-seq analysis), and that the gene expression signature of this hybrid cell cluster was recapitulated in the bulk RNA-seq analysis of adherent cells, suggesting that this phenotype is present in the majority of MSC-adherent ALL cells.

Parallel functional genomic and biochemical assays provided additional insights into the causes and consequences of this adherence-induced EMT transition. First, the focused CRISPR-Cas9 genome editing screen identified ITGB1 (as part of the VLA-4 heterodimer) as a mediator of this adhesion. Second, blockade of ITGB1 and VLA-4 resensitized ALL cells adhered to MSCs to DEX. Lastly, the knockdown of EMT TFs TWIST1 and SNAI2 alleviated the chemoresistance in MSC-adherent ALL cells, indicating that EMT drives the acquisition of this chemoresistant phenotype in adherent ALL cells.

Biochemical assays were notable for showing activation of WNT, NOTCH1, TGFβ, RTK, and cytokine signaling pathways known to drive EMT in MSC-adherent leukemia cells. However, only the inhibition of WNT/β-catenin and cytokine signaling reduced the viability of leukemic cells interacting with MSCs, indicating that the NOTCH1, TGFβ, and RTK signaling pathways may have other functions (e.g., ECM remodeling) or may be dispensable during EMT. Our findings are consistent with prior data showing that the inhibition of the WNT/β-catenin pathway sensitized leukemic cell lines co-cultured with stromal cell lines and improved overall survival in a mouse model of leukemia cell line, but high concentrations of XAV-939, a stimulator of β-catenin degradation by stabilization of Axin, were required.^62^

These assays also revealed that only a subset of these EMT-associated activated pathways were suitable for targeting to achieve therapeutic benefits. Indeed, we discovered that β-catenin-mediated transcriptional activity is increased in hybrid ALL cells exhibiting THY1 expression, and specific inhibition of the β-catenin-CBP complex, which is known to promote self-renewal, diminished the survival and drug resistance of PDX ALL cells adhered to MSCs *ex vivo* and reduced leukemic burden of NALM6 xenograft model *in vivo*. This indicates that hybrid, MSC-adherent ALL cells utilize β-catenin-CBP complex-mediated transcription and are vulnerable to inhibition of β-catenin-CBP interaction.

### Limitations of the study

The *ex vivo* and *in vivo* studies described here have several limitations. Our studies solely focused on the interaction of ALL with MSCs within BME, although other components of the BME, including vascular niche cells, are also important for chemoresistance. Additionally, we used NALM6 cells in many assays due to their facility for experimental manipulation such as transduction, but importantly, results were corroborated in studies with primary or PDX cells and co-culture with primary MSCs. While it is possible that culture conditions (lower cell density and reduced serum) in co-culture might affect leukemic adherence, we demonstrated that the culture media type did not play a major role in leukemic adhesion to MSCs. Further, while *in vivo* PDX models are extensively used to test antileukemic drug efficacy in ALL, such models are of limited utility in studying the interaction between human leukemic cells and human BME because NSG mice lack human BME. Furthermore, although PRI-724 is very effective as a single drug and in combination with DEX *in vitro*, due to its short half-life *in vivo*, it should be viewed as a tool compound demonstrating potential utility of inhibiting this pathway. However, our data demonstrate that deregulation of leukemic cell biology by physical interaction with the BME is an important determinant of resistance to therapy. These results extend our findings of genotype (IKZF1 alteration)-determined perturbation of adhesion and stemness as a driver of drug resistance in *BCR::ABL1* leukemia to show that ALL-stromal interactions are important across the spectrum of ALL genetic subtypes. Moreover, these data identify therapeutic approaches that may ameliorate this stromal-mediated resistance.

## STAR★METHODS

### RESOURCE AVAILABILITY

#### Lead contact

Further information and requests for resources and reagents should be directed to and will be fulfilled by the Lead Contact, Charles Mullighan (charles.mullighan@stjude.org). An MTA will be required for all requested materials.

#### Materials availability

This study did not generate new unique reagents. Information on reagents used in this study in available in the key resources table.

#### Data and code availability

- Bulk and single-cell RNA-seq data from cell lines have been deposited at the Gene Expression Omnibus (GEO) under accession number GEO: GSE200039 and are publicly available as of the date of publication. Bulk RNA-seq data from human primary patient and normal samples have been deposited at the European Genome-phenome Archive (EGA) under accession number EGA: EGAS00001006120.
- This paper does not report original code.
- Any additional information required to reanalyze the data reported in this paper is available from the lead contact upon request.

### EXPERIMENTAL MODEL AND STUDY PARTICIPANTS DETAILS

#### Human subjects

Leukemia samples were obtained from subjects were enrolled on St. Jude Children’s Research Hospital (SJCRH) and ECOG-ACRIN Cancer Research Group studies. This study was approved by institutional review boards at SJCRH and ECOG-ACRIN and informed consent was obtained from parents, guardians, or patients, and assent from the patient, as appropriate.

#### Mouse studies

All mice work was carried out according to Office of Laboratory Animal Welfare guidelines and was approved by the Institutional Animal Care and Use Committee of St Jude Children’s Research Hospital.

#### Cell lines

All the cell lines were confirmed as Mycoplasma spp. free using the Universal Mycoplasma Detection Kit (American Type Culture Collection, Manassas, VA). Cell lines were maintained in RPMI 1640 medium supplemented with 10% FBS (Hyclone), Antibiotic (1X, Gibco), and GlutaMAX (1X, Gibco) and incubated in a 37°C humidity-controlled incubator with 5% CO_2_. Each cell line identity was confirmed by STR profiling using PowerPlex Fusion System (Promega) and confirmed using ATCC or DSMZ STR database.

HEK293T cells (ATCC, Manassas, VA) were cultured in Dulbecco’s Modified Eagle’s Medium (DMEM) (Lonza) supplemented with 10% fetal bovine serum (FBS) (Cytiva), and 100 U/mL penicillin, 100 μg/mL streptomycin, 2 mM glutamine (P/S/G) (Thermo Fisher Scientific). 697, NALM6, and REH were maintained in Roswell Park Memorial Institute (RPMI) 1640 medium supplemented with 10% FBS and P/S/G. SUP-B15 and MHH-CALL-2 were cultured in RPMI 1640 medium supplemented with 20% FBS and P/S/G. For either coculture with human BM-MSC or mono-culture for experiments, all leukemic cell lines were cultured in α-modified Minimum Essential Medium (α-MEM) (Sigma-Aldrich) supplemented with 10% FBS, and P/S/G. All cells were incubated in a humidified incubator at 37°C with 5% CO_2_.

#### Generation of primary human BM-MSC

Human BM-MSC were prepared by available protocol^32^ with some modification of procedure. BM aspirates from special hematology laboratory of SJRCH were mixed with Lymphoprep (STEMCELL Technologies) followed by centrifugation at 800 x g for 20 min. Middle layer which contains mononuclear cells were collected and plated into tissue culture plate with α-MEM supplemented with 10% FBS, and P/S/G. After the initial 48 h of incubation, the medium containing nonadherent cells were removed and adherent cells were washed twice with phosphate-buffered saline (PBS) to get rid of the remaining floating cells. The adherent cells were further expanded until the outgrowth of colonies (colony unit forming fibroblasts, CFU-F) was observed. These cells are considered passage 0 (p0).

#### Cell culture of primary BM-MSC and hTERT-MSC

Primary BM-MSC were cultured in α-MEM supplemented with 10% FBS, and P/S/G. hTERT-MSC^27^ were maintained in RPMI 1640 medium supplemented with 10% FBS, P/S/G and 1.0 μM Hydrocortisone (Sigma-Aldrich).

#### *In vitro* differentiation of primary BM-MSC

For osteogenic differentiation, BM-MSC were pre-cultured in 6 well plates using α-MEM supplemented with 10% FBS, and P/S/G until reaching confluence and were replaced with 2mL of complete MesenCult Osteogenic Stimulatory Medium which was made of MesenCult Osteogenic Differentiation Kit (Human) (STEMCELL Technologies) per well. Once multilayering had been observed, aspirated medium and replaced with 2mL of complete Medium with β-glycerophosphate (Sigma-Aldrich). Medium was changed every 3 days for 2–3 weeks. Osteogenic differentiation is visualized by staining with Alizarin Red S (Sigma).

For chondrogenic differentiation, we followed methods reported in Reinisch et al.^32^ For optimized 3-dimensional (3-D) chondrogenic differentiation, BM-MSCs were centrifuged onto 0.4 μm pore size membranes of transwell inserts (24 well plates, Corning), and cultivated in DMEM medium containing 40 μg/mL L-proline, 25 μg/mL L-ascorbic acid 2-phosphate, insulin-transferrin-sodium selenite plus linoleic-bovine serum albumin (BSA) (ITS+1 Liquid Media Supplement) (Sigma-Alrich), 10^−7^ M dexamethasone (Selleckchem), sodium pyruvate (Thermo Fisher Scientific) and 10 ng/mL TGF-β3 (PeproTech). After 28 days of differentiation, formalin-fixed and paraffin-embedded tissue was stained with Alcian blue, Toluidine Blue and Safranin O (Sigma).

Adipogenic differentiation, BM-MSC were pre-cultured in 6 well plates using α-MEM supplemented with 10% FBS, and P/S/G until reaching confluence and were replaced with 2mL of complete MesenCult Adipogenic Differentiation Medium made of MesenCult Adipogenic Differentiation Kit (Human) (STEMCELL Technologies) per well. Media was changed every 3 days for 10–20 days. Adipogenic differentiation was visualized by staining with Oil Red O staining (Sigma).

#### RNA-seq

hTERT-MSC were cultured until confluent. 5 ALL cell lines including 697, MHH-CALL-2, NALM6, REH, and SUP-B15 were co-cultured with hTERT-MSC for two days. After two days, the cells exist in supernatant from the dish were collected as Nonadh ALL cells, whereas Adh ALL cells bound to MSC were dissociated by TrypLE treatment after washing with PBS. Cells were stained with fluorescent antibody for CD19 and CD90, then sorted by flow cytometry (minimum 99%). The total RNAs from the Nonadh and Adh cells were isolated using Qiagen kit. The RNA-seq libraries were prepared using the Illumina TruSeq Stranded Total RNA library preparation kit and sequenced on HiSeq 4000 (Illumina) with 100-bp paired-end setting. For B-ALL patient samples (Table S3), primary MSC were used for co-culture. Since there is limited numbers of sorted cell from patient samples, a low-input RNA library preparation kit (NuGen Ovation V2) was used to generate the RNA-seq libraries, followed by sequencing on HiSeq 4000 (Illumina) with 100-bp paired-end setting.

The sequencing libraries from cell lines were mapped to the GRCh37 human genome using StrongArm mapping pipeline^65^ and gene read counts were generated using HTSeq (version 0.6.1) and differential express analysis was done using R/Limma-Voom.

For sequencing libraries from patient samples, the reads were mapped to the GRCh37 human genome reference by STAR (version 2.5.3a).^66^ Gene expression was quantified using RSEM^67^ (v1.3.0) against Ensembl gene annotation (v75) (http://www.ensembl.org/). Differential expression between Adh and Nonadh patient cells was done using paired moderated t test (R/limma-Voom).^68^ In addition, gene fusions and B-ALL subtype analyses was performed as described in Gu et al.^2^ for the RNA-seq data from patient samples.

Gene set enrichment analysis (GSEA) was done using Pre-ranked GSEA^34^ with ratios from individual cell line comparisons, combined cell line comparison or paired patient sample comparison. Multiple gene sets collected in MSigDB were explored.

#### Single cell RNA-seq

To unravel cell heterogeneity in co-cultured cells, 1.5 × 10^6^ NALM6 cells were co-cultured with BM-MSC in T175 flask for two days. The cells exist in supernatant from the dish were collected as Nonadh NALM6 and Adh NALM6 with BM-MSC were collected after dissociation with TrypLE. In comparison, NALM6 and MSC were also collected and subjected to single cell RNAseq using 10x Chromium Next GEM Single Cell 3′ v3. The Cell Ranger Single-Cell Software Suite (version 1.1.0) was used to perform sample demultiplexing, barcode processing, single-cell gene counting. Raw base call (BCL) files were demultiplexed using the Cell Ranger mkfastq pipeline into sample-specific FASTQ files that were then processed individually using the Cell Ranger count pipeline with default setting. Each sample was analyzed using Seurat^69^ package (version 4.0.6) in R. The tSNE plots were generated using res = 0.4. Differential expression analysis between NALM6 and MSC was performed using FindMarkers function in Seurat with default parameters (logfc.threshold = 0.25, test.use = "wilcox", min.pct = 0.1). Only the significant genes expressed in >50% of cells in one group (pct.1 > 0.5 from FindMarkers output) and <10% in the other (pct.2 < 0.1) were retained as marker genes, resulting in 178 for NALM6 and 244 for MSC. These markers were further aggregated to calculate enrichment scores for NALM6 and MSC using AddModuleScore function in Seurat and plot the heatmap in Figure 5B.

### METHOD DETAILS

#### Cell viability assay

To measure selectively leukemic cells co-cultured with BM-MSC, ALL cells were modified to express a previously described yellow fluorescent protein (YFP) and firefly luciferase (ffLuc) by transduction with a vector (vCL20SF2-Luc2a-YFP).^63^ Marked ALL with vCL20SF2-Luc2a-YFP were plated into the 96 well plate with or without MSC and co-cultured for 24 h. Subsequently, drugs were treated for 72 h and cell viability was determined by measuring relative luminescence units (RLU) using CellTiter-Glo Luminescent Cell Viability Assay (Promega, Madison, WI) with GloMax Discover (Promega). Three technical replicates were used for each of three biological replicate experiments. Cell Cultures and maintained in RPMI 1640 with 10% FBS and 2 mM GlutaMAX. To assess drug synergisms of between two drugs, SynergyFinder 2.0 was used.^64^

#### CRISPR screening

Two leukemia cell lines were used to study genes responsible for adhesion by targeted CRISPR KO library: B-progenitor ALL cell lines NALM-6 (*IGH::DUX4*) and 697 (*TCF3::PBX1*. Guide RNAs (gRNAs) targeting the adhesome (http://www.adhesome.org/components.html), TGFβ pathway, and non-template control were prepared and cloned into LentiCRISPRv2 (Cas9+eGFP+gRNAs) plasmid backbone which expresses Cas9 and gRNAs by the Center for Advanced Genome Engineering (CAGE) at SJRCH.

The sgRNA sequences for 284 coding genes were compiled from GeCKO^70^ and Brunello^71^ CRISPR libraries (Table S5). Duplicate gRNAs were removed, and off-target analysis was performed. Ten gRNA sequences per gene were selected with unique target sites; these sequences were supplemented with gRNA sequences designed using the Broad GPP sgRNA design tool CRISPick (https://portals.broadinstitute.org/gpp/public/analysis-tools/sgrna-design. Non-targeting control gRNAs were added and comprise 10% of the total library. The final library design contained 3105 sgRNAs targeting 284 genes and 283 non-targeting control gRNAs. The resulting oligo library was designed according to the GeCKO protocol^72^ and synthesized (Twist Bioscience). Library amplification and Gibson Assembly into the pLentiCRISPRv2 backbone (Addgene #82416) was performed according to the prior method.^73^ The python scripts count_spacers.py^72^ and calc_auc_v1.1.py^74^ were used to validate library representation and quality control. The validated library was subsequently used to produce viral particles. Production and titration of lentiviral vectors were performed as described previously.^75^

Optimal infection conditions were determined for each cell line in order to achieve 20–60% infection efficiency, corresponding to a multiplicity of infection (MOI) of ~0.5–1. RetroNectin (TaKaRa)-coated plates were preloaded with virus supernatant by centrifugation for 2 h, 1500xg, at 4°C and then 2x10^6^ leukemic cells were added and cultured for 2 days at CO_2_ incubator. Infections were performed in three biological replicates per cell line using cells and viral amount that yielded around 40% infection efficiency to achieve target representation of ~500 cells per sgRNA. At 48 h post transduction, GFP positive cells were isolated using FACSAria III cell sorter (BD Biosciences). Thereafter, 0.7-1.1x10^6^ cells were harvested as the baseline counts for the control, and 2x10^6^ cells were co-cultured with MSC in T175 flask considering that each sgRNA, on average, was represented ~500 times. Adherent and non-adherent cells were collected after 48 h of co-culture. Cells harvested at each time point were subjected to genomic DNA/RNA extraction using AllPrep DNA/RNA Mini Kit. Genomic DNA was quantitated by Nanodrop 2000 (Thermo Fisher scientific). PCR amplification was performed using protocols described in the Broad Genome Perturbation Portal (https://portals.broadinstitute.org/gpp/public/resources/protocols). Single-end, 100 cycle sequencing was performed on a NovaSeq 6000 (Illumina) by the Hartwell Center for Genome Sequencing Facility at SJRCH. CRISPR KO screens were analyzed using Mageck-Vispr/0.5.6.^76^

#### Cell adhesion assay

NALM6 were preincubated with isotype antibody or integrin β1 antibodies (P5D2 and AIIB2) for 30 min on ice and these mixtures were plated into confluent MSC plate. Four hours after coculture, Nonadh and Adh NALM6 were counted. In addition, cell adhesion assays were performed in the presence of integrin α4β1 inhibitor (BIO-1211), α5β1 inhibitor (ATN-161), or combination of both with same procedure above. NALM6 WT or ITGB1 knockout (KO) were co-cultured with confluent MSC for 4 h and then Nonadh and Adh NALM6 were counted. When using YFP-positive NALM6 cells, Nonadh cells were washed out with PBS three time, the level of Adh cells bound either MSC or fibronectin (50 μg/mL) was determined by measuring RLU using CellTiter-Glo Luminescent Cell Viability Assay (Promega, Madison, WI) with GloMax Discover (Promega).

#### Cell aggregation assay

Cells were seeded at a density of 2x10^5^ cells/2mL into 6 well non-tissue plate (triplicate) and incubated for 24 h before being imaged at 10x using an Eclipse TS100 light microscope. The number of cell clusters consisting of more than four cells was counted and area (pixel per square) of cell cluster was measured by ImageJ.

#### Cell migration assay

Migration assays were performed using the QCM 24-Well Fluorimetric Cell Migration Assay kit (Cat No: ECM 509). To facilitate cell migration, MSC were plated at lower chamber and cultured for 4 days. YFP^+^ NALM6 and Adh YFP^+^ NALM6 cell suspensions were prepared, containing 1 × 10^6^ cells/mL in serum-free α-MEM. Then, 300 μL of the prepared suspension was added to each insert. Next, 500 μL of α-MEM supplemented with 10% FBS and P/S/G was added to the lower chamber, and the plates were incubated for 24 h at 37°C in a CO_2_ incubator. Afterward, the cells/media from the top side of the insert were carefully removed by pipetting out the remaining cell suspension. Then, the invasion chamber insert was removed and the image of the YFP^+^ NALM6 cells that migrated from top chamber and subsequently bound to MSC was captured by fluorescence microscopy and the number of migrated YFP^+^ NALM6 was analyzed ImageJ.

#### Cell invasion assay

Invasion assays were performed using the QCM 24-Well Fluorimetric Cell Migration Assay kit (Cat No: ECM 554). To facilitate cell invasion, MSC were plated at lower chamber and cultured for 4 days. YFP^+^ NALM6 and Adh YFP^+^ NALM6 cell suspensions were prepared, containing 1 × 10^6^ cells/mL in serum-free α-MEM. Then, 300 μL of the prepared suspension was added to each insert. Next, 500 μL of α-MEM supplemented with 10% FBS and P/S/G was added to the lower chamber, and the plates were incubated for 24 h at 37°C in a CO_2_ incubator. Afterward, the cells/media from the top side of the insert were carefully removed by pipetting out the remaining cell suspension. Then, the invasion chamber insert was removed and the image of the YFP^+^ NALM6 cells that migrated from upper chamber through the ECM layer and subsequently bound to MSCs was captured by fluorescence microscopy and the number of migrated YFP^+^ NALM6 was analyzed ImageJ.

#### ECM array

NALM6 cell line was plated at cell density of 1 × 10^6^ cells/mL onto the ECM Select Array Kit (Advanced Biomatrix) containing 36 different single ECM protein or combinations, each in 9 micro-spots. Twenty-four hours incubation, the ECM Select Array Kit was washed with PBS to remove non-specifically bound cells and was imaged at 4x magnification using an Eclipse TS100 light microscope (Nikon, Melville, NY).

#### Electron microscopy

For electron microscopy, MSC were plated in 8 well Lab-Tek Permanox slide chambers and cultured for 48 h and then NALM6 cells were added into MSC-cultured chamber and cultured for 48 h. Nonadh NALM6 cells were washed out by PBS. Adherent NALM6 with MSC were fixed by EM fixative composed of 4% paraformaldehyde and 0.5% glutaraldehyde in 0.1M phosphate buffer (PB) for 10 min at room temperature. After removal of fixative and immediate washing with PB, co-cultured sample was quenched with 0.1% sodium borohydride in PB. Integrin beta 1 is visualized by labeling with anti-Integrin beta 1 antibody (P5D2) from Abcam, an ultrasmall gold conjugated goat anti-mouse antibody (Aurion) and silver enhancement (Aurion R-Gent SE-EM). Following labeling and silver enhancement, samples were osmicated, contrasted *en bloc* with uranyl acetate, dehydrated and infiltrated in EmBed-812 resin (Electron Microscopy Sciences). Fully infiltrated samples were polymerized at 60°C for 48 h. Samples were sectioned at 70nm on a Leica UC-7 ultramicrotome and imaged in a ThermoFisher Scientific TF-20 operating at 80kV equipped with an AMT NanoSprint15 MkII imaging system.

#### Generation of stable NALM6 cell line expressing lentivector co-expressing dscGFP and luciferase in response to TCF/LEF

pGreenFire1-TCF/LEF (EF1α-neo) Lentivector (System Biosciences) were packaged into replication-incompetent, amphotropic lentiviral particles by transient transfection of HEK293T cells with pHDM-G, CAG4-RTR2, and CAG-KGP1-1R. NALM6 was transduced with lentivirus in the presence of RetroNectin (Takara) for 48 h. For the selection of a stable cell line expressing pGreenFire1-TCF/LEF (EF1α-neo) NALM6 (NALM6-TCF/LEF) cells were grown in RPMI 1640 medium supplemented with 10% FBS and P/S/G plus 400 μg/mL G418. Selection media was refreshed every 3 days until 14 days.

#### Immunoblotting

Cells are lysed in RIPA buffer (Sigma-Aldrich) containing 150 mM NaCl, 1.0% IGEPAL CA-630, 0.5% sodium deoxycholate, 0.1% SDS, 50 mM Tris, pH 8.0 supplemented with proteosome and phosphatase inhibitors for 1hr on ice. Protein samples (15 μg of lysate) electrophoresed through 4–12% NuPAGE Bis-Tris gels (Life Technologies) at 200 V for 60 min. Blots were probed with anti-fibronectin, anti-TWIST, anti-SLUG, anti-β-catenin, anti-Cleaved NOTCH1, anti-pSMAD3, anti-pSMAD1/5/8, anti-pNFκB p65, anti-pSTAT3 and anti-Actin.

#### Immunofluorescence

Cytospins of NALM6 alone and co-cuture samples of NALM6 with BM-MSC were fixed with 4% paraformaldehyde and washed with PBS, followed by 1 h incubation in a 3% BSA blocking buffer. After removal of blocking buffer, Cells were permeabilized by incubation with 0.1% Triton X-100 in PBS for 10 min at room temperature and then incubated for 1 h with primary anti-ITGB1 antibody (P5D2, Abcam) diluted in 3% NGS/0.1% Triton X-100/PBS. Slides were washed three times in PBS, and then incubated for 1 h in 3% NGS/0.1% Triton X-100/PBS containing goat anti-rabbit Alexa Fluor 568 (Invitrogen, Carlsbad, CA). Slides were washed three times in PBS and mounted with ProLong Diamond Antifade Mountant with 4′-6-diamidino-2-phenylindol (DAPI) (ThermoFisher Scientific). All steps were carried out at room temperature. Images were captured using a Nikon C2 confocal fluorescence microscope and analyzed using NIS Elements software.

#### Cytokine array assay

Cytokine profiling was performed using Proteome Profiler Array Human (XL) Cytokine Array kit (R&D Systems), which detects 105 human cytokines simultaneously. We used cell culture supernatants collected from BM-MSC only, NALM6 only, and co-culture of NALM6 and BM-MSC, which were cultured for two days. Cytokine arrays were incubated overnight at 4°C with 500 μL of cell culture supernatant on a rocking platform. Following incubation with antibody detection cocktail, antibody conjugation, and recommended washes, membranes were washed, incubated with the antibody cocktail diluted for 1 h, washed, and incubated with streptavidin-HRP for 30 min, and finally treated with chemiluminescent reagent mix; membranes were exposed to film and imaged. Arbitrary values of cytokine abundance were calculated as integrated densities of each dot plot normalized by the reference spots. Integrated densities were measured using the ImageJ software (v1.50i).

#### Tunneling nanotube-mediated vesicle transfer

Donor cells were labeled with Vybrant DiD Cell-Labeling Solution (Thermo Fisher Scientific), 5 μL per ml of cell culture medium for 15 min at 37°C, washed three times. Afterward, cells were seeded in co-culture with acceptor cells in 6-well cell culture plates (1 × 10^5^ MSC cells plus 1 × 10^5^ YFP-positive NALM6 cells) and co-cultured for 24 h. To physical separate the interaction between NALM6-YFP and MSC, MSC and YFP-positive NALM6 cells were plated in the lower and upper chambers of a transwell with 0.4 μm pore polyester membrane insert (Corning). After 24 h, acceptor cells received DiD-labeled components were analyzed by flow cytometry analysis.

#### Flow cytometric analysis

Flow antibodies listed in REAGENT or RESOURCE were used for flow cytometric analysis. Cellular fluorescence data were collected for three technical replicates on BD LSRFortessa cytometer (Becton Dickinson Poland) using DIVA software (BD Biosciences, Franklin Lakes, NJ), and analyzed with FlowJo vX.0.6 (Tree Star, Inc., Ashland, OR).

For intracellular phosphorylation analysis, NALM6 cells were co-cultured with BM-MSC for indicated times. Cells were dissociated by TrypLE and neutralized by adding of culture medium followed by centrifugation. Cells were resuspended in PBS and fixed with same volume of BD Fixation/Permeabilization solution at 37°C for 10 min. Fixation was stopped by addition of medium followed by centrifugation. Cells were resuspended in 2% FBS-PBS. After Fc block, cells were stained with FITC Mouse Anti-Human CD19 (SJ25C1) and PE Mouse Anti-Human CD90 (5E10) antibodies. After permeabilization with 1x BD Perm/Wash Buffer for 30 min on ice and cells were stained with Fluorochrome conjugated antibodies or Phospho-primary antibodies followed by staining with Alexa Fluor 647 Goat Anti-Rabbit IgG H&L.

#### siRNA knock-down

For knockdown experiments with small interfering RNA (siRNA), NALM6 cells mixed with 300 nM SilencerSelect siRNAs (Thermo Fisher Scientific) against ITGA4 (1: s7544, 2: s7545), ITGA5 (1: s7548, 2:s7549), TWIST1 (1: s14524, 2: s529181), SNAI2 (1: s13128, 2: s13129) or non-targeting control siRNA (Cat.# 4390843) were electroporated in Resuspension Buffer R with the Neon transfection system using 3 pulse at 1410V for 10 ms each. 48h after nucleofection, target protein expression was determined by flow cytometry or immunoblotting.

#### Cell cycle analysis

Cells were fixed and permeabilized in 250 μL of fixation/permeabilization solution from BD Cytofix/Cytoperm kit for 20 min at 4°C and washed in 1 mL of BD Perm/Wash buffer. Then, cells were stained with APC anti-human Ki-67 antibody for 20 min at 4° C and washed 2 mL of BD Perm/Wash buffer. Cells were incubated in staining buffer in the presence of 7-AAD at 1:40 dilution for 20 min at room temperature (BD). The cells were analyzed with BD LSRFortessa.

#### CRISPR/Cas9 genome editing

To achieve gene knockout (KO) of *ITGB1*, NALM6 cells were mixed with ribonucleoprotein (RNP) complexes that consist of 10pmole of purified Cas9 nuclease duplexed with 90 pmol of multi-guide chemically modified *ITGB1* synthetic single guide RNA (sgRNA) (predesigned Gene Knockout Kit v2, Synthego). The cells were then resuspended in 12μL of R Buffer (Thermo Fisher Scientific) for electroporation in the Neon Transfection System (Thermo Fisher Scientific). Cells were loaded in 10μL tips and electroporated according to the manufacturer’s instructions using the setting of 1410 V, 10ms width, and 3 pulse. Following electroporation, cells were recovered in RPMI (Thermo Fisher Scientific) containing 20% FBS (Thermo Fisher Scientific) for 24 h before undergoing another round of electroporation to increase the knockout efficiency. Cells were then harvested for single cell sorting 24 h later. Knockout efficiencies of all clones were analyzed by flow cytometry.

#### Proteome profiling by tandem mass tag mass spectrometry (TMT-MS)

Proteome profiling was performed using a previously published protocol^77^ with slight modifications. Cell pellets and media were resuspended in lysis buffer (50 mM HEPES, pH 8.5, 8.0 M urea and 0.5% sodium deoxycholate) and protein concentrations were determined by a Coomassie stained short gel with BSA as a standard.^78^ Approximately 100 μg of protein per sample were proteolyzed using Lys-C (Wako) at an enzyme-to-substrate ratio of 1:100 (w/w) for 3 h at 21°C. Following this the samples were diluted with 50 mM HEPES (pH 8.5) to a final 2.0 M urea. Disulfide bonds were reduced with DTT (5 mM and 45 min incubation at 21°C) and alkylated with iodoacetamide (20 mM and 45 min incubation at 21 °C in the dark). Proteins were further digested with trypsin (Promega) at an enzyme-to-substrate ratio of 1:50 (w/w) overnight at 21°C. Finally, the enzyme digestions were terminated by adding formic acid (FA) to 1% and samples were clarified at 21,000*g*, desalted on tC18 SepPak solid-phase extraction cartridges (Waters), and dried by SpeedVac.

The purified TMT-labeled peptides were resuspended in 50 μL 100 mM HEPES (pH 8.5) and labeled with 10-plex TMT reagents (Thermo Fisher Scientific, 10 μg/μL in 100% acetonitrile (ACN), 1:2 (w/w) peptide-TMT ratio, 1 h at 21°C). The labeling reactions were quenched by adding hydroxylamine to 0.3% (v/v), and labeled peptides were combined equally, acidified by adding FA to 1% (v/v), desalted and dried.

The pooled peptides were resuspended in 10 mM ammonium formate (pH 8.5) buffer and fractionated on an offline HPLC (Agilent 1220) using basic pH reverse phase liquid chromatography (pH 8.5, XBridge C18 column, 4.6 mm × 25 cm, 3.5 μm particle size, Waters) with a 180 min gradient of 15%–50% Buffer B [90% ACN, 10 mM ammonium formate (pH8.5)] and collected into 90 concatenated fractions. Fractions were dried in SpeedVac and resuspended in 5 μL 5% FA.

A QE-HF (Thermo Fisher Scientific) mass spectrometer connected in-line to a Dionex Ultimate 3000 ultra-high pressure liquid chromatography (UHPLC) system was used for LC-MS/MS analysis. Peptides were separated on a CoAnn column (75 μm × 15 cm, 1.9 μm C18, column at 50°C) using a 60 min gradient of 12%–56% Buffer B [67% ACN/2.5% DMSO/0.1% FA] at 0.25 μL/min flow rate. The mass spectrometer was operated in data-dependent mode with a survey scan in Orbitrap (60,000 resolution, 410–1600 m/z, 1 × 10^6^ AGC target, 50 ms maximal ion time), followed by 20 data-dependent MS2 scans in Orbitrap (60,000 resolution, scan range starting from 120 m/z, 1 × 10^5^ AGC target, ~105 ms maximal ion time, 32 HCD normalized collision energy, 1.0 m/z isolation window with 0.2 m/z offset,15 s dynamic exclusion, charge state screening enabled to reject precursor charge states that were unassigned, +1, or >+4).

MS raw files were searched against Uniprot HUMAN protein database using our in-house developed JUMP pipeline.^79^ Database search parameters included precursor and fragment ion mass tolerance 20 ppm, fully tryptic with maximal 2 missed cleavages, and maximal 3 modification sites per peptide. TMT10 tags on Lys residues and N-termini (+229.162932 Da) and carbamidomethylation of Cys (+57.02146 Da) were set as static modifications while Met oxidation (+15.99492 Da) were set as dynamic modifications. Peptide-spectrum match (PSM) were filtered to reduce the protein false discovery rate (FDR) to below 1% based on the target-decoy strategy using reversed database.^80^ TMT-based quantification and statistical analysis were performed based on our previous method.^81^ TMT quantification data quality was evaluated by comparing positive control protein intensities with Western blot results and by utilizing statistical methods (PCA or unsupervised clustering) to determine outliers or sample mislabeling. Protein fold change and p values of different comparisons were calculated based on protein intensities. Differential expression analysis was performed based on our previous method,^82^ by applying different combinations of cutoff threshold [i.e., FDR (Benjamini-Hochberg corrected p) < 0.05] based on the positive protein list (proteins known to be dysregulated in the comparisons).

#### *In vivo* pharmacokinetic (PK) studies

Female NSG (NOD.Cg-Prkdcscid Il2rgtm1Wjl/SzJ) mice aged 8 to 12 weeks were dosed by intraperitoneal injections with single dose daily for 3 days of PRI-724 at 20 mg/kg and 40 mg/kg, respectively. PRI-724 was suspended in 4.2% DMSO+95.8% PBS. After last injections of PRI-724, blood samples (3 mice per group) were collected at 2, 6 and 24 h by retro-orbital eye bleeding. Plasmas were prepared by centrifugation for 10 min at 1,500x*g* using a refrigerated centrifuge. Concentration of PRI-724 and its active form C-82 in plasma was determined by LC-MS.

#### Cellular senescence activity assay

We followed instruction of Cell Meter Cellular Senescence Activity Assay Kit (AAT Bioquest). Briefly, Xite Red beta-D-galactopyranoside working solution were prepared by adding 10 μL Xite Red beta-D-galactopyranoside stock solution dissolved in DMSO to 1 mL of assay buffer. Cells were washed with PBS, incubated with 100 μL Xite Red beta-D-galactopyranoside working solution for 30 min at 37°C, 5% CO_2_ incubator. After removing the working solution, cells were washed with PBS and resuspended in the assay buffer (Component B) and monitored the fluorescence intensity (MFI) with flow cytometer using 575/26 nm filter set.

#### *In vivo* preclinical studies

Female NSG mice aged 8 to 12 weeks were inoculated by tail vein injection with 1 × 10^6^ YFP^+^ NALM6 cells, which were modified to express YFP and ffLuc by transduction with a vector (vCL20SF2-Luc2a-YFP). Leukemia burden was determined by bioluminescence imaging using a Xenogen IVIS-200 system and Living Image software (Caliper Life Sciences). Treatment was started 7 days post injection (luminescence averaged ~4x10^7^ photons per second) and continued until 24 days post injection. Mice were randomized following injection 6 days post injection and were treated with either vehicle, dexamethasone (4 mg/L) alone, PRI-723 alone (40 mg/kg per day) via intraperitoneal injection, or combination of dexamethasone and PRI-724. Dexamethasone was given daily in drinking water with tetracycline (1 g/L; Sigma-Aldrich, St. Louis, MO), and half of each week the water contained Sulfamethoxazole (600 mg/L) and trimethoprim (120 mg/L; from Aurobindo).^83^ Mice were sacrificed when they became moribund.

### QUANTIFICATION AND STATISTICAL ANALYSIS

Analyses of functional data were performed using GraphPad Prism Version 8.0 (GraphPad, La Jolla, CA). All data are presented as mean ± SD. Differential expression between Adh and Nonadh patient cells was done using paired moderated t test (R/limma-Voom). Enriched genes in 11 patient Adh ALL samples compared to Nonadh ALL samples (Log2(Adh/Nonadh) > 1, FDR<0.05) are shown. The significance of the drug effect on cell viability was assessed by two-way ANOVA with a Sidak adjustment for multiple comparisons between control and drug-treated samples. Analysis of drug synergisms between two drugs was performed using SynergyFinder 2.0. ZIP synergy score is used for the synergy score (Less than −10, antagonistic; from −10 to 10, additive; larger than 10, synergistic). Statistical testing of BLI was performed using two-way ANOVA with Tukey’s multiple comparisons test. For comparison between two groups, data were analyzed with a two-tailed Student’s t-test. A p value of less than 0.05 was considered significant. *p ≤ 0.05, **p ≤ 0.005, ***p ≤ 0.0005. All the statistical details of the experiments can be found in the figure legends.

## Supplementary Material

1

2

3

4

5

6

## Figures and Tables

**Table:** KEY RESOURCES TABLE

REAGENT or RESOURCE	SOURCE	IDENTIFIER
Antibodies		
Anti-Integrin Beta1, clone AIIB2 (Azide Free) Antibody	Sigma-Aldrich	Cat# MABT409, RRID:AB_2893323
Integrin beta 1 antibody [P5D2]	Abcam	Cat# ab24693, RRID:AB_448230
Human VCAM-1/CD106 MAb (Clone BBIG-V1)	R and D Systems	Cat# BBA5, RRID:AB_356958
Fibronectin antibody	Abcam	Cat# ab2413, RRID:AB_2262874
Slug (C19G7) Rabbit mAb	Cell Signaling Technology	Cat# 9585, RRID:AB_2239535
Twist antibody [Twist2C1a] - ChIP Grade	Abcam	Cat# ab50887, RRID:AB_883294
Snail (C15D3) Rabbit mAb	Cell Signaling Technology	Cat# 3879, RRID:AB_2255011
TCF8/ZEB1 (D80D3) Rabbit mAb	Cell Signaling Technology	Cat# 3396, RRID:AB_1904164
ZEB2 (E6U7Z) Rabbit mAb	Cell Signaling Technology	Cat# 97885, RRID:AB_2934315
β-Catenin (D10A8) XP^®^ Rabbit mAb	Cell Signaling Technology	Cat# 8480, RRID:AB_11127855
Cleaved Notch1 (Val1744) (D3B8) Rabbit mAb	Cell Signaling Technology	Cat# 4147, RRID:AB_2153348
Recombinant Anti-Smad3 (phospho S423 + S425) antibody [EP823Y]	Abcam	Cat# ab52903, RRID:AB_882596
Phospho-Smad1 (Ser463/465)/Smad5 (Ser463/465)/Smad9 (Ser465/467) (D5B10) Rabbit mAb	Cell Signaling Technology	Cat# 13820, RRID:AB_2493181
Ultrasmall gold conjugated Goat Mouse Ab (Aurion)	Electron Microscopy Sciences	Cat# 25121
Phospho-NF-κB p65 (Ser536) (93H1) Rabbit mAb	Cell Signaling Technology	Cat# 3033, RRID:AB_331284
Phospho-Stat3 (Tyr705) (D3A7) XP Rabbit mAb	Cell Signaling Technology	Cat# 9145, RRID:AB_2491009
Mouse Anti-beta-Actin Monoclonal Antibody, Unconjugated, Clone AC-74	Sigma-Aldrich	Cat# A5316, RRID:AB_476743
Alexa Fluor^®^ 647 Anti-Smad2 (pS465/ pS467)/Smad3 (pS423/pS425) antibody	BD Biosciences	Cat# 562696, RRID:AB_2716578
Mouse Anti-Human Akt, phospho (Ser473) Monoclonal Antibody, Alexa Fluor??647 Conjugated	BD Biosciences	Cat# 560343, RRID:AB_1645397
Goat Anti-Rabbit IgG H&L (Alexa Fluor^®^ 647)	Abcam	Cat# ab150079, RRID:AB_2722623
APC-H7 Anti-Human CD45 Antibody	BD Biosciences	Cat# 560178, RRID:AB_1645479
PE Anti-Human CD14 Antibody	BD Biosciences	Cat# 561707, RRID:AB_10924593
APC Anti-Human HLA-DR Antibody	BD Biosciences	Cat# 560896, RRID:AB_10563218
FITC Anti-Human CD34 Antibody	BD Biosciences	Cat# 560942, RRID:AB_10562559
FITC Anti-Human CD90 Antibody	BD Biosciences	Cat# 555595, RRID:AB_395969
FITC Anti-Human CD19 Antibody	BioLegend	Cat# 363008, RRID:AB_2564171
PE Anti-Human CD90 Antibody	BioLegend	Cat# 328110, RRID:AB_893433
APC Anti-Human CD90 Antibody	BD Biosciences	Cat# 559869, RRID:AB_398677
BV605 Anti-Human CD73 Antibody	BD Biosciences	Cat# 563199, RRID:AB_2738063
FITC Anti-Human CD105 Antibody	BD Biosciences	Cat# 561443, RRID:AB_10714629
APC Anti-Human CD105 Antibody	BD Biosciences	Cat# 562408, RRID:AB_11154045
PerCP-Cy^™^5.5 Anti-Human CD19 Antibody	BD Biosciences	Cat# 340950, RRID:AB_400191
PerCP-Cy^™^5.5 Anti-Human CD90 Antibody	BD Biosciences	Cat# 561557, RRID:AB_10712762
PE Anti-Human CD49d (Integrin alpha 4) Antibody	Thermo Fisher Scientific	Cat# 12-0499-42, RRID:AB_10717245
BV421 Anti-Human CD49E (Integrin α5 chain) Antibody	BD Biosciences	Cat# 740077, RRID:AB_2739840
APC Anti-Human CD51 (Integrin alpha V) Antibody	BioLegend	Cat# 327913, RRID:AB_2876633
Bacterial and virus strains		
One Shot^™^ Stbl3^™^ Chemically Competent *E. coli*	Thermo Fisher Scientific	Cat# C737303
Biological samples		
Human Bone Marrow Aspirates	SJCRH	N/A
Human B cell Acute Lymphoblastic Leukemia Patient Samples	ECOG-ACRIN Cancer Research Group (ECOG-ACRIN)	SJALL054548, SJALL054550, SJALL054551, SJALL054554, SJALL054563, SJALL055623, SJALL055625, SJALL055626, SJALL054535, SJALL057947, SJALL057948
Patient-derived xenograft (PDX) cells derived from B-ALL patients	This paper	SJBALL022611_R4, SJBALL047370_R1, SJBALL055854_X1, SJBALL021896_D1, SJBALL265_D1, SJBALL020589_D1
Chemicals, peptides, and recombinant proteins		
Dexamethasone	Sigma-Aldrich	Cat# D4902
ATN-161	Selleckchem	Cat# S8454
XAV-939	Selleckchem	Cat# S1180
LF3	Selleckchem	Cat# S8474
LY2157299	Selleckchem	Cat# S2230
BAY 11-7082	Selleckchem	Cat# S2913
Stattic	Selleckchem	Cat# S7024
PRI-724	Selleckchem	Cat# S8968
LY411575	Selleckchem	Cat# S2714
APC Annexin V	BioLegend	Cat# 640941
Fibronectin	Advanced BioMatrix	Cat# 5050
7-AAD	BioLegend	Cat# 420403
Hydrocortisone	Sigma-Aldrich	Cat# H0888
Lymphoprep^™^	STEMCELL Technologies	Cat# 07851
Minimum Essential Medium Eagle (MEME)	Sigma-Aldrich	Cat# M4526
DMEM 4.5 g/L Glucose w/o L-Gln w/Phenol Red	Lonza	Cat# BE12-614F
RPMI 1640 Medium, no glutamine	Thermo Fisher Scientific	Cat# 21870092
RetroNectin^®^	TaKaRa	Cat# T100B
L-Proline	Sigma-Aldrich	Cat# P5607
HyClone Fetal Bovine Serum (FBS)	Cytiva	Cat# SH30071.03HI
L-Ascorbic Acid 2-Phosphate Sesquimagnesium Salt Hydrate	Sigma-Aldrich	Cat# A8960
ITS+1 Liquid Media Supplement (100×)	Sigma-Aldrich	Cat# I2521
Recombinant Human TGF-β3	Peprotech	Cat# 100-36E
Ascorbic Acid	STEMCELL Technologies	Cat# 72132
β-Glycerophosphate Disodium Salt Hydrate	Sigma-Aldrich	Cat# G9422
Toluidine blue O	Sigma-Aldrich	Cat# 1.15930
Alcian blue Solution	Sigma-Aldrich	Cat# 1.01647
Alizarin Red Staining Solution	Sigma-Aldrich	Cat# TMS-008
Safranin O Solution	Sigma-Aldrich	Cat# HT90432
Oil Red O Staining Solution	Sigma-Aldrich	Cat# 1.02419
Silver enhancement (Aurion R-Gent SE-EM)	Electron Microscopy Sciences	Cat# 25521
EmBed-812 resin	Electron Microscopy Sciences	Cat# 14120
Sodium Pyruvate (100 mM)	Thermo Fisher Scientific	Cat# 11360070
Penicillin-Streptomycin-Glutamine (100X)	Thermo Fisher Scientific	Cat# 10378016
TrypLE^™^ Express Enzyme (1X), No Phenol Red	Thermo Fisher Scientific	Cat# 12604013
SuperSignal^™^ West Femto Maximum Sensitivity Substrate	Thermo Fisher Scientific	Cat# 34096
ProLong^™^ Diamond Antifade Mountant with DAPI	Thermo Fisher Scientific	Cat# P36962
Critical commercial assays		
MesenCult^™^ Osteogenic Differentiation Kit (Human)	STEMCELL Technologies	Cat# 05465
MesenCult^™^ Adipogenic Differentiation Kit (Human)	STEMCELL Technologies	Cat# 05412
CellTiter-Glo^®^ Luminescent Cell Viability Assay	Promega	Cat# G7571
Gene Knockout Kit v2 - human - ITGB1 - 1.5 nmol	Synthego	N/A
BD Cytofix/Cytoperm^™^	BD Biosciences	Cat# 554714
Vybrant^™^ DiD Cell-Labeling Solution	Thermo Fisher Scientific	Cat# V22887
Cell Meter^™^ Cellular Senescence Activity Assay Kit *Red Fluorescence*	AAT Bioquest	Cat# 23007
AllPrep DNA/RNA Mini Kit (50)	Qiagen	Cat# 80204
QCM^™^ 24-Well Fluorimetric Cell Migration Assay	Sigma-Aldrich	Cat# ECM 509
Neon^™^ Transfection System 100 μL Kit	Thermo Fisher Scientific	Cat# MPK10025
Neon^™^ Transfection System 10 μL Kit	Thermo Fisher Scientific	Cat# MPK1025
Deposited data		
RNA-seq data from human primary patient and normal samples	This paper	EGA: EGAS00001006120
RNA-seq data from cell lines	This paper	GEO: GSE200039
Experimental models: Cell lines		
NALM6	ATCC	Cat# CRL-3273
REH	ATCC	Cat# CRL-8286
SUP-B15	ATCC	Cat# CRL-1929
HEK293T	ATCC	Cat# CRL-3216
NALM6 marked with YFP-Luc2 (YFP-positive NALM6)	This paper	N/A
hTERT-MSC	Mihara et al.^27^	N/A
Experimental models: Organisms/strains		
Mouse: NOD.Cg-Prkdcscid Il2rgtm1Wjl/SzJ (NSG)	The Jackson Lab	Cat# 005557
Oligonucleotides		
ITGB1 PCR forward primer: AAACTGGTCA GAGTTTGCATAAAGC	This paper	N/A
ITGB1 PCR reverse primer: AAGGACTT TTCAAAGGGAAATTTTG	This paper	N/A
ITGB1 sequencing primer: GTAAACACG CAGGTATTCACAGAG	This paper	N/A
ITGB1 guide_#1: U*G*C*UGUUCCUUU GCUACGGU *Synthego modified EZ Scaffold	Synthego	N/A
ITGB1 guide_#2: G*A*U*GACAUAGA AAAUCCCAG *Synthego modified EZ Scaffold	Synthego	N/A
ITGB1 guide_#3: A*U*U*UAGACAUUUUUACAGGA *Synthego modified EZ Scaffold	Synthego	N/A
Non-targeting control Silencer^®^Select siRNA	Thermo Fisher Scientific	Cat# 4390843
ITGA4 Silencer^®^Select siRNA (ITGA4-1)	Thermo Fisher Scientific	Cat# s7544
ITGA4 Silencer^®^Select siRNA (ITGA4-2)	Thermo Fisher Scientific	Cat# s7545
ITGA5 Silencer^®^Select siRNA (ITGA5-1)	Thermo Fisher Scientific	Cat# s7548
ITGA5 Silencer^®^Select siRNA (ITGA5-2)	Thermo Fisher Scientific	Cat# s7549
TWIST1 Silencer^®^Select siRNA (TWIST1-1)	Thermo Fisher Scientific	Cat# s14524
TWIST1 Silencer^®^Select siRNA (TWIST1-2)	Thermo Fisher Scientific	Cat# s529181
SNAI2 Silencer^®^Select siRNA (SNAI2-1)	Thermo Fisher Scientific	Cat# s13128
SNAI2 Silencer^®^Select siRNA (SNAI2-2)	Thermo Fisher Scientific	Cat# s13129
Recombinant DNA		
pGreenFire1-TCF/LEF (EF1 α-neo) Lentivector	System Biosciences	Cat# TR013PA-N
vCL20SF2-Luc2a-YFP	Iacobucci et al.^63^	N/A
pLentiCRISPRv2	Addgene	Cat# 82416
Software and algorithms		
Morpheus	https://software.broadinstitute.org/morpheus	RRID:SCR_017386
FlowJo 10.7.2	https://flowjo.com/	RRID:SCR_008520
EnrichR	https://maayanlab.cloud/Enrichr/	RRID:SCR_001575
SynergyFinder 2.0	Ianevski et al.^64^	https://synergyfinder.fimm.fi
ImageJ 1.50i	https://imagej.nih.gov/ij/download.html	RRID:SCR_003070
GraphPad Prism	https://www.graphpad.com:443/	RRID:SCR_002798
Other		
Adhesome	Winograd-Katz et al.^37^; Zaidel-Bar et al.^38^	http://www.adhesome.org/components.html
Amersham Hyperfilm MP	Cytiva	Cat# 28906846

## Highlights

- Acute lymphoblastic leukemia interaction with mesenchymal stromal cells drives drug resistance
- Interaction is mediated by integrin beta 1 and drives an EMT phenotype
- Activated WNT/β-catenin signaling may be targeted to reverse drug resistance
