# Supplementary material for: Stromal-induced epithelial-mesenchymal transition induces targetable drug resistance in acute lymphoblastic leukemia
Source: Cell Rep. Author manuscript; Available in PMC 2023 Sep 27. (PMC10529385; doi:10.1016/j.celrep.2023.112804)
Supplement: 1 [file NIHMS1920517-supplement-1.pdf]

Cell Reports, Volume *42*

Supplemental information

Stromal-induced epithelial-mesenchymal transition induces targetable drug resistance in acute lymphoblastic leukemia

Chun Shik Park, Hiroki Yoshihara, Qingsong Gao, Chunxu Qu, Ilaria Iacobucci, Pankaj S. Ghate, Jon P. Connelly, Shondra M. Pruett-Miller, Ben Wagner, Camenzind G. Robinson, Ashutosh Mishra, Junmin Peng, Lei Yang, Zoran Rankovic, David Finkelstein, Selina Luger, Mark Litzow, Elisabeth M. Paietta, Nikhil Hebbar, M. Paulina Velasquez, and Charles G. Mulligan

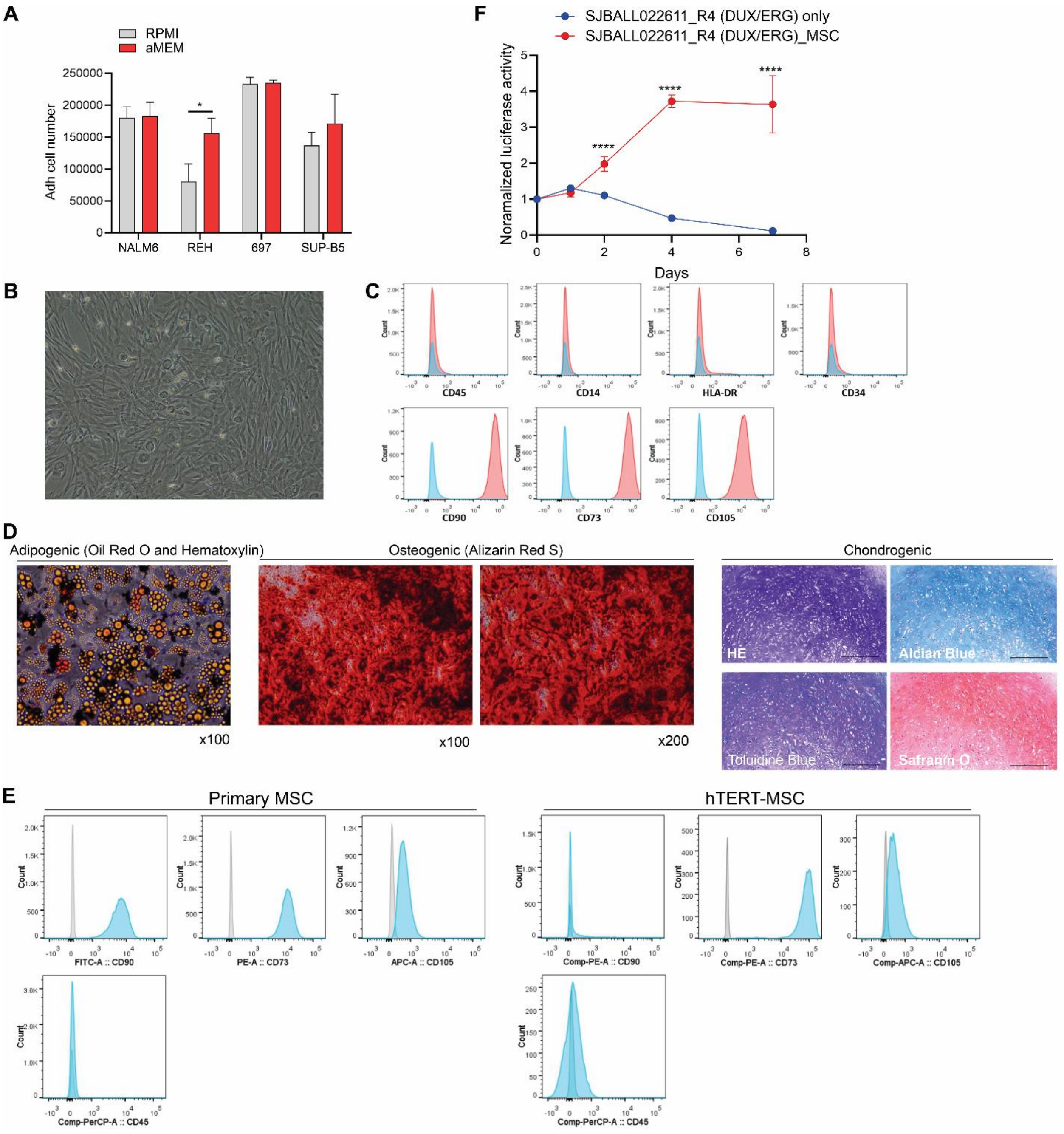

Figure S1. Phenotypic and functional properties of primary MSC and hTERT-MSC, and effects of MSC coculture on PDX cells, related to Figure 1. (**A**) Effect of media on leukemia cell line adhesion to primary MSC. Cell lines were plated onto MSC-coated plates in the presence of either RPMI or α-MEM for 4 hours, and the number of adherent (Adh) cells was quantified (n=3). Data, mean ± SD. (**B**) Primary MSC is plastic adherent. (**C**) Primary MSC express CD90, CD73 and CD105, and lack expression of CD45, CD14, HAL-DR, and CD34 surface molecules. (**D**) Primary MSC retain ability to differentiate to osteoblasts, adipocytes and chondroblasts *in vitro*. (**E**) hTERT-MSC express extremely low levels of CD90 compared to primary MSC. (**F**) Effect of MSC coculture on PDX cell proliferation. SJBALL022611_R4 (*DUX4*-rearranged) PDX cells marked with YFP/luciferase were cultured in either alone or with MSC (n=3). Cell proliferation was determined by luciferase activity. Data, mean ± SD. Statistical testing was performed using 2-tailed Student's t test, ****$p < 0.0001$, *$p < 0.05$.

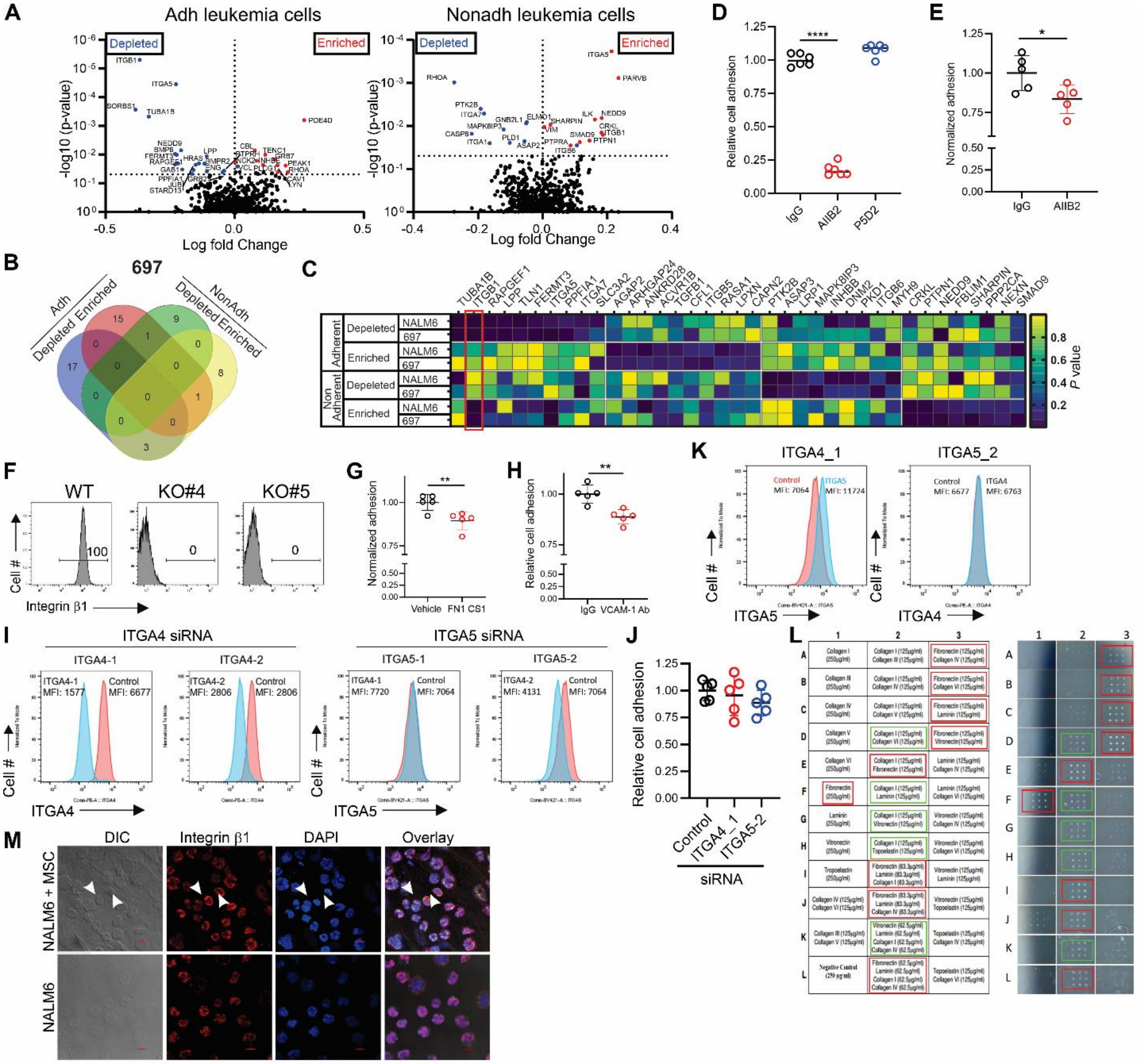

Figure S2. Characterization of interaction between ALL cells and primary MSC, related to Figure 3. (**A**) Volcano plots displaying the $\log_{10}$ fold change on the X axis and Log_{10} P value on the Y axis for all sgRNAs identified in Adh 697 and Nonadh 697. (**B**) Venn diagram showing number of genes which are either depleted or enriched genes in Nonadh and Adh 697. (**C**) Summary diagram showing enriched or depleted genes in either Adh or Nonadh ALL cells. (**D**) Cell adhesion of NALM6 cells on fibronectin in the presence of either AIIB2 or P5D2 (20 μg/ml; n=6). Data, mean with individual values. (**E**) Cell adhesion of PDX (SJBALL022611_R4) cells to MSCs in the presence of IgG or AIIB2 Ab (20 μg/ml; n=5). After 24 hours, Adh PDX cells were quantified. Data, mean ± SD. (**F**) Flow cytometric analysis of integrin β1 in ITGB1 knock out clones. (**G, H**) Cell adhesion of NALM6 cells onto MSC in the presence of FN1 CS1 peptide (50 μg/ml) and VCAM-1 Ab (20 μg/ml; n=5). Data, mean ± SD. (**I**) The knockdown efficiency of siRNAs targeting ITGA4 (1 and 2) and ITGA5 (1 and 2) in NALM6 cells. (**J**) Cell adhesion assay using either ITGA4 or ITGA5 knock down NALM6 cells (n=5). Data, mean with individual value. (**K**) Flow cytometric analysis of ITGA5 in ITGA4 knock down NALM6 and ITGA4 in ITGA5 knock down NALM6. (**L**) ECM array to screen ECM responsible for NALM6 binding. Red box: NALM6 binding to fibronectin and fibronectin-containing combinations. Green box: NALM6 binding to ECM combinations without fibronectin. (**M**) Immunofluorescence imaging of integrin β1 in NALM6 or NALM6 co-cultured with MSC. Statistical testing was performed using 2-tailed Student's t test, ****p <0.0001, **p <0.005, *p < 0.05.

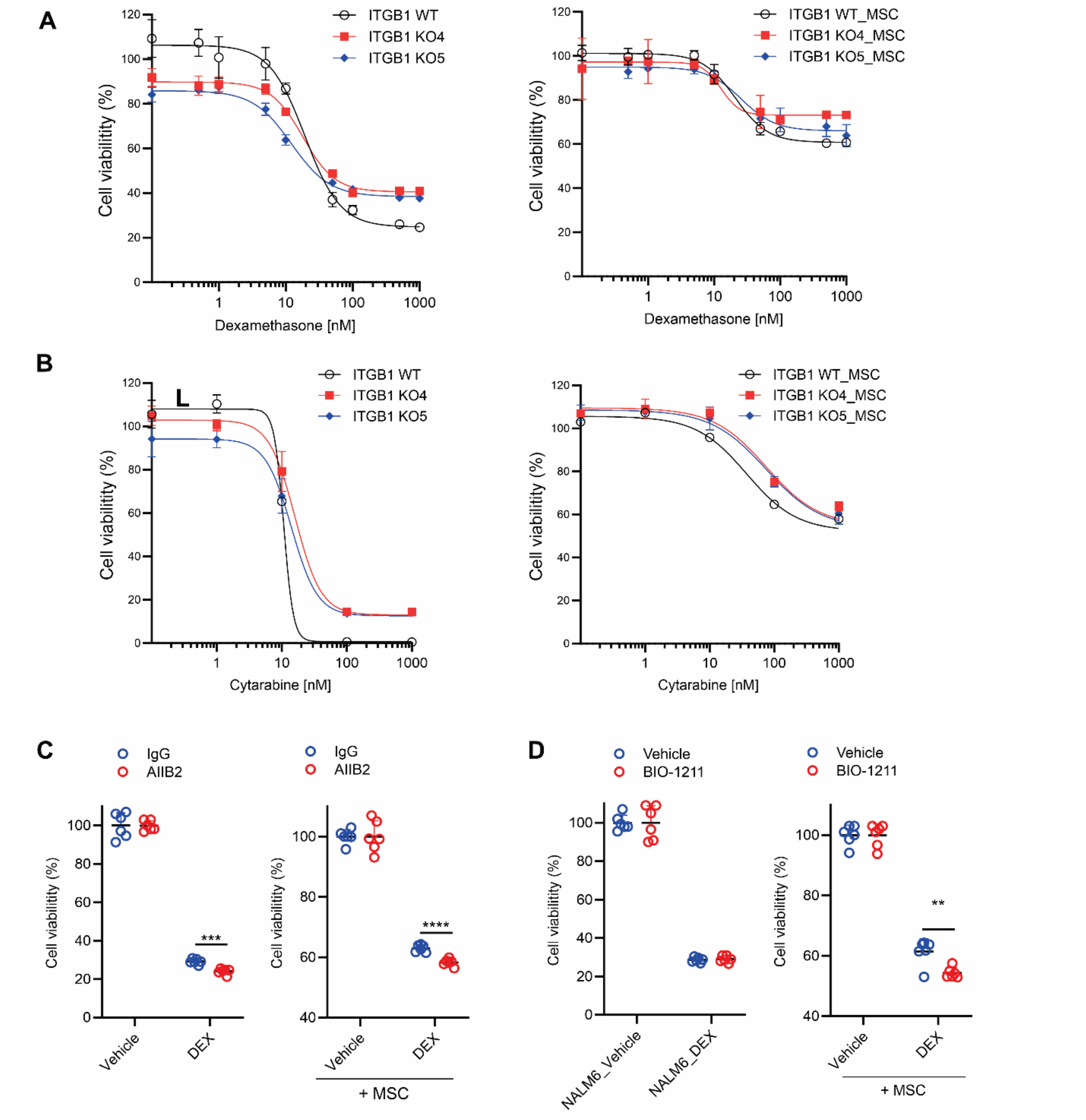

Figure S3. Blockade of ITGB1 increases the drug sensitivity of NALM6 co-cultured with MSC, related to Figure 3. (**A**) Sensitivity of integrin β1 knockout (ITGB1 KO) NALM6 to DEX in the presence or absence of MSC (n=3). Data, mean ± SD. (**B**) Sensitivity of integrin β1 knockout (ITGB1 KO) NALM6 to cytarabine in the presence or absence of MSC (n=3). Data, mean ± SD. (**C**) Cell viability of NALM6 co-cultured with MSC in the presence of either 20 μg/ml integrin β1 blocking antibody (AIIB2) or IgG (n=6). (**D**) Cell viability of NALM6 co-cultured with MSC in the presence of 50 μM BIO-1211, the blocker of integrin α4β1 (n=6). Data, individual value with mean. Statistical testing was performed using 2-tailed Student's t test, ****p <0.0001, ***p <0.0005, **p <0.005.

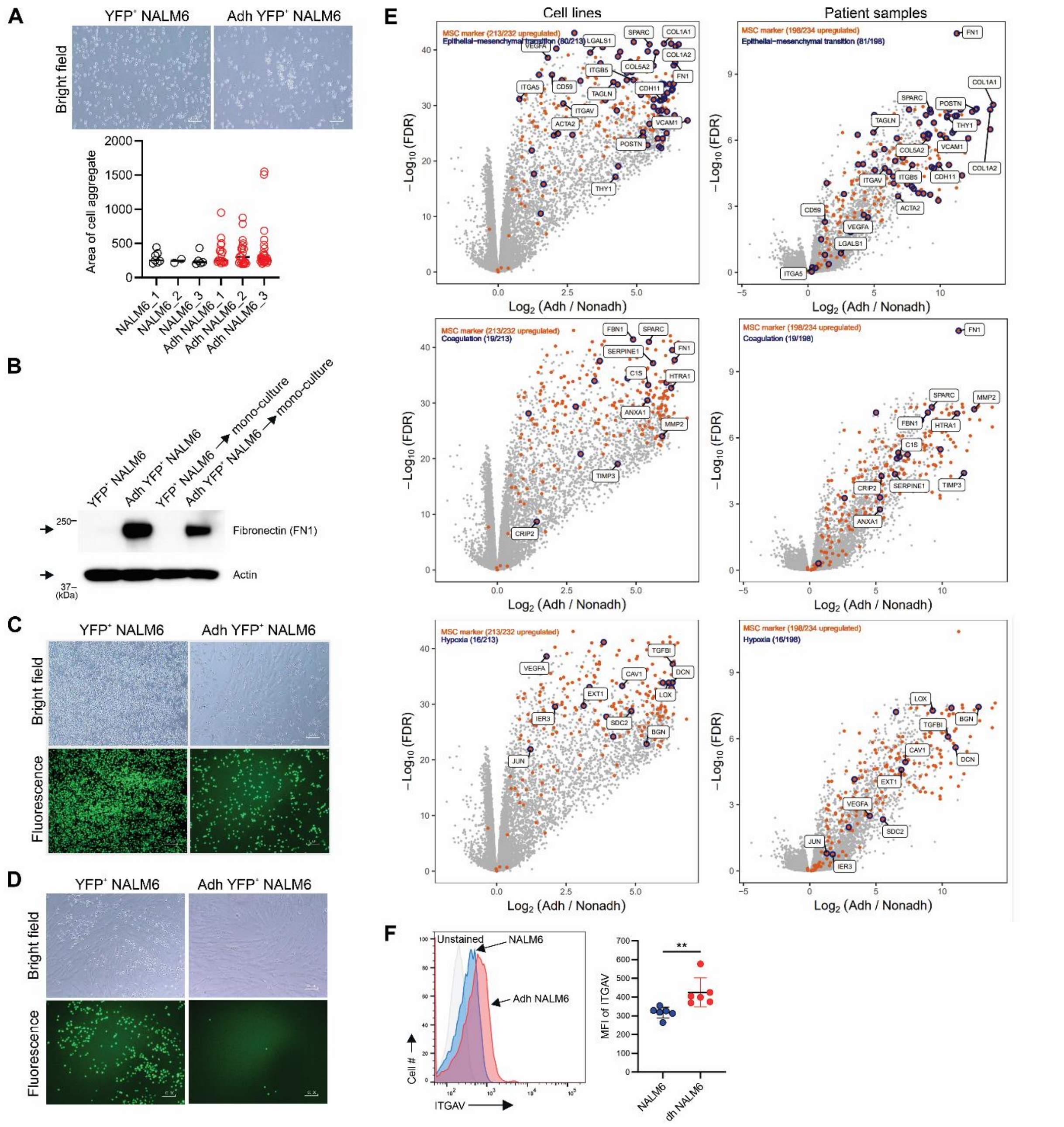

Figure S4. Functional properties of Adh ALL cells; comparison of scRNA-sec MSC signature genes and upregulated genes in Adh vs Nonadh B-ALL cells from bulk RNA-seq analysis; and flow cytometric analysis of ITGAV in Adh ALL cells, related to Figures 4 and 5. (**A**) Cell aggregation assay of Adh YFP+ NALM6 (n=3). Area (pixel per square) was measured by ImageJ. Area higher than 200 (equivalent to 4 cells) is displayed. (**B**) Immunoblotting of mono-cultured YFP+ NALM6 and Adh YFP+ NALM6 and additional mono-cultured samples. (**C**) Cell migration assay (n=3). The photos of YFP+ NALM6 that migrated from upper chamber and subsequently bound to MSCs was captured by fluorescence microscope. (**D**) Cell invasion assay (n=3). The photo of YFP+ NALM6 that migrated from upper chamber through the ECM layer and subsequently bound to MSC. (**E**) Orange color indicates that MSC signature genes are listed in DEGs of bulk RNA-seq and blue circle represents genes of indicated hallmark pathway. Left panels show MSC signature genes and DEGs of bulk RNA-seq and right panels show MSC signature genes and DEGs of bulk RNA-seq. (**F**) Flow cytometry of ITGAV in NALM6 and Adh NALM6 (n=6). MFI: mean fluorescence. Data, mean ± SD.

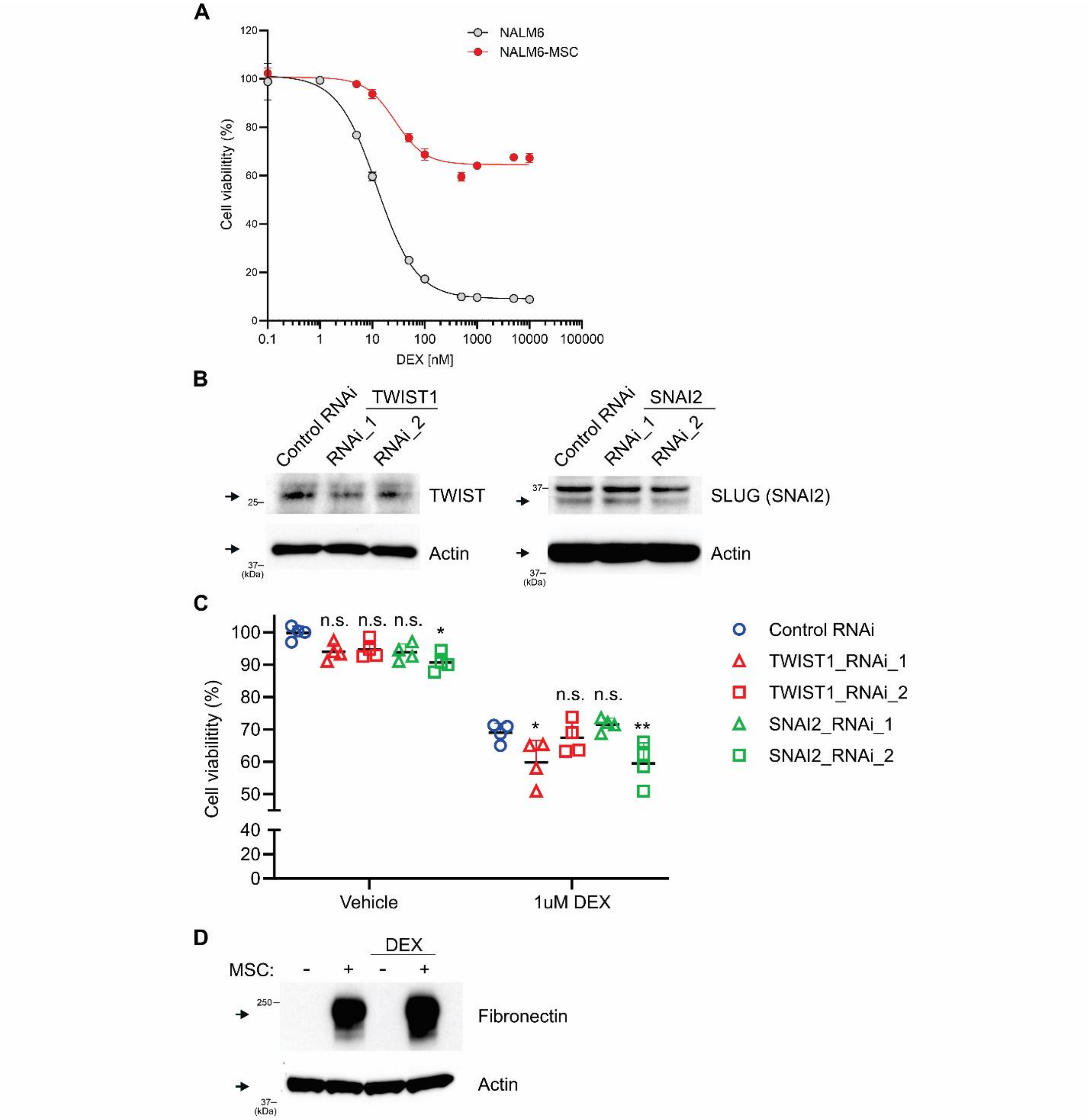

Figure S5. MSC-mediated DEX resistance in NALM6 cells cocultured with MSC, and the effect of TWIST1 or SNAI2 knockdown in NALM6 cells co-cultured with MSC on DEX treatment, related to Figure 6. (**A**) Sensitivity of NALM6 to DEX in the presence or absence of MSC (n=3). Data, mean ± SD. (**B**) Immunoblotting analysis of levels of TWIST1 and SLUG (SNAI2) proteins in NALM6 cells that were nucleofected with either TWIST (left panel) or SNAI2 siRNAs (right panel) at 48 hours after co-cultured with MSC. (**C**) Cell viability assay of YFP$^+$ NALM6 cells treated with siRNAs against control, TWIST1, or SNAI2 (n=4). NALM6 cells nucleofected with control, TWIST1, or SNAI2 siRNAs were seeded onto an MSC plate and incubated for one day, followed by treatment with either vehicle or 1 μM DEX for three days. Data, individual value with mean. Statistical testing was performed using Two-way ANOVA with Dunnett's multiple comparisons test comparisons test, **p <0.005, *p < 0.05. (**D**) EMT state enhanced by Adh cells under treatment of DEX. YFP$^+$ NALM6 cells were mono-cultured or co-cultured with MSC and treated with either vehicle or 50 nM DEX. YFP$^+$ NALM6 cells were purified by cell sorting and subjected to immunoblotting analysis for fibronectin.

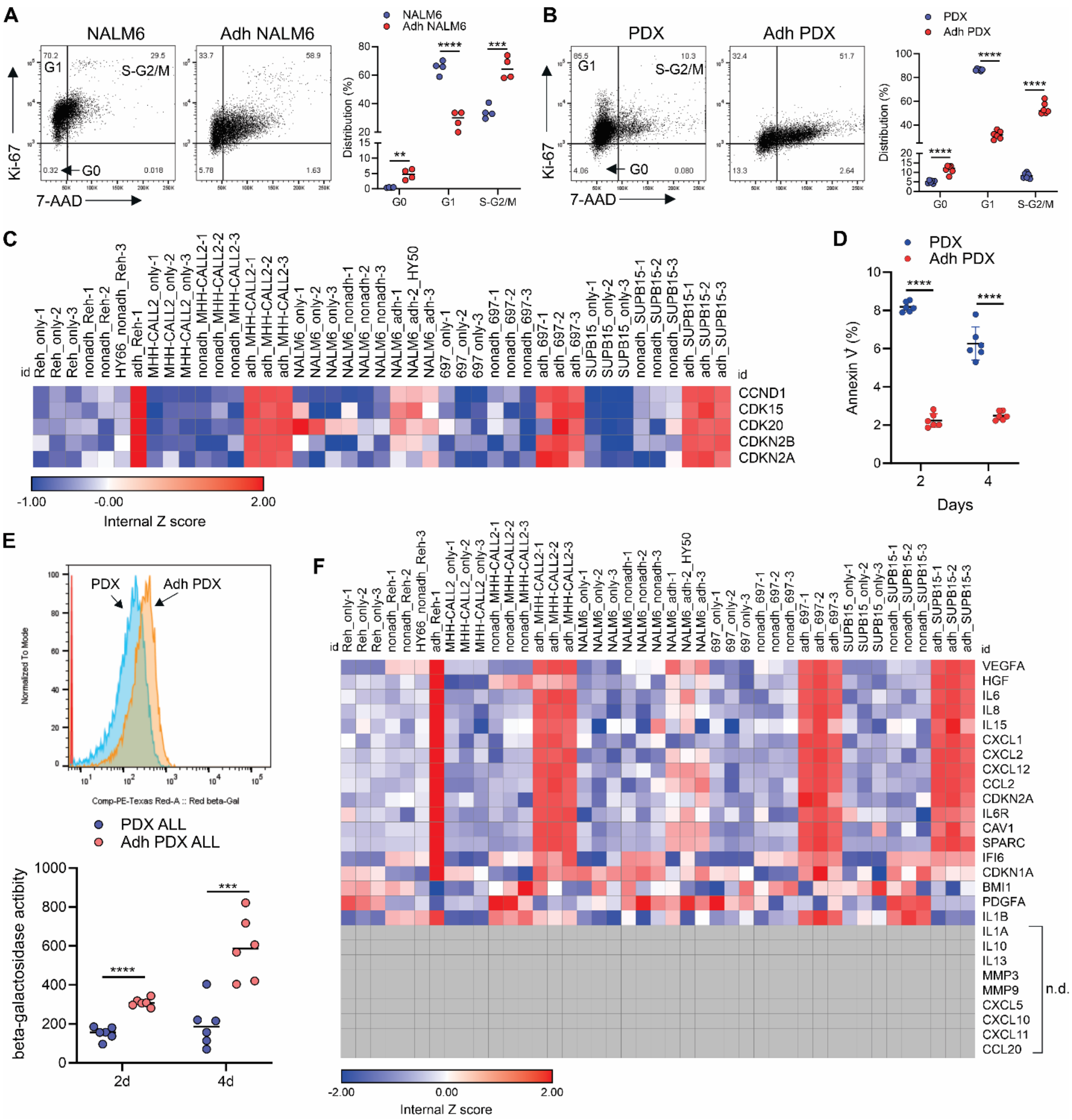

Figure S6. Impact of MSC on leukemic cells in co-culture, related to Figure 6. (**A**) Cell cycle in NALM6 and Adh NALM6 was determined by Ki-67 and 7-AAD staining (n=4). Data, individual value with mean. (**B**) Cell cycle of PDX cells was determined by Ki-67 and 7-AAD staining. (**C**) Deregulation of cell cycle control genes in Adh cell lines. (**D**) The effect of MSC co-culture on PDX cell survival (n=6). Apoptotic cells of PDX (SJBALL022611_R4) cells was determined by Annexin V$^+$ staining after gating of YFP$^+$ cells. Data, mean ± SD. (**E**) Measurement of cell senescence levels by β-galactosidase activity in PDX alone (blue) and Adh PDX (orange; n=6). Left top panel shows the representative histogram of PDX alone and Adh PDX after 2 days culture. The bottom left panel displays the cumulative β-galactosidase activity data from PDX and Adh PDX samples cultured for two and four days. Data, individual value with mean. (**F**) Senescence-associated secretory phenotype (SASP) genes are enriched in Adh cell lines. n.d.: not detectable. Statistical testing was performed using 2-tailed Student's t test, ****p <0.0001, ***p <0.0005, **p <0.005.

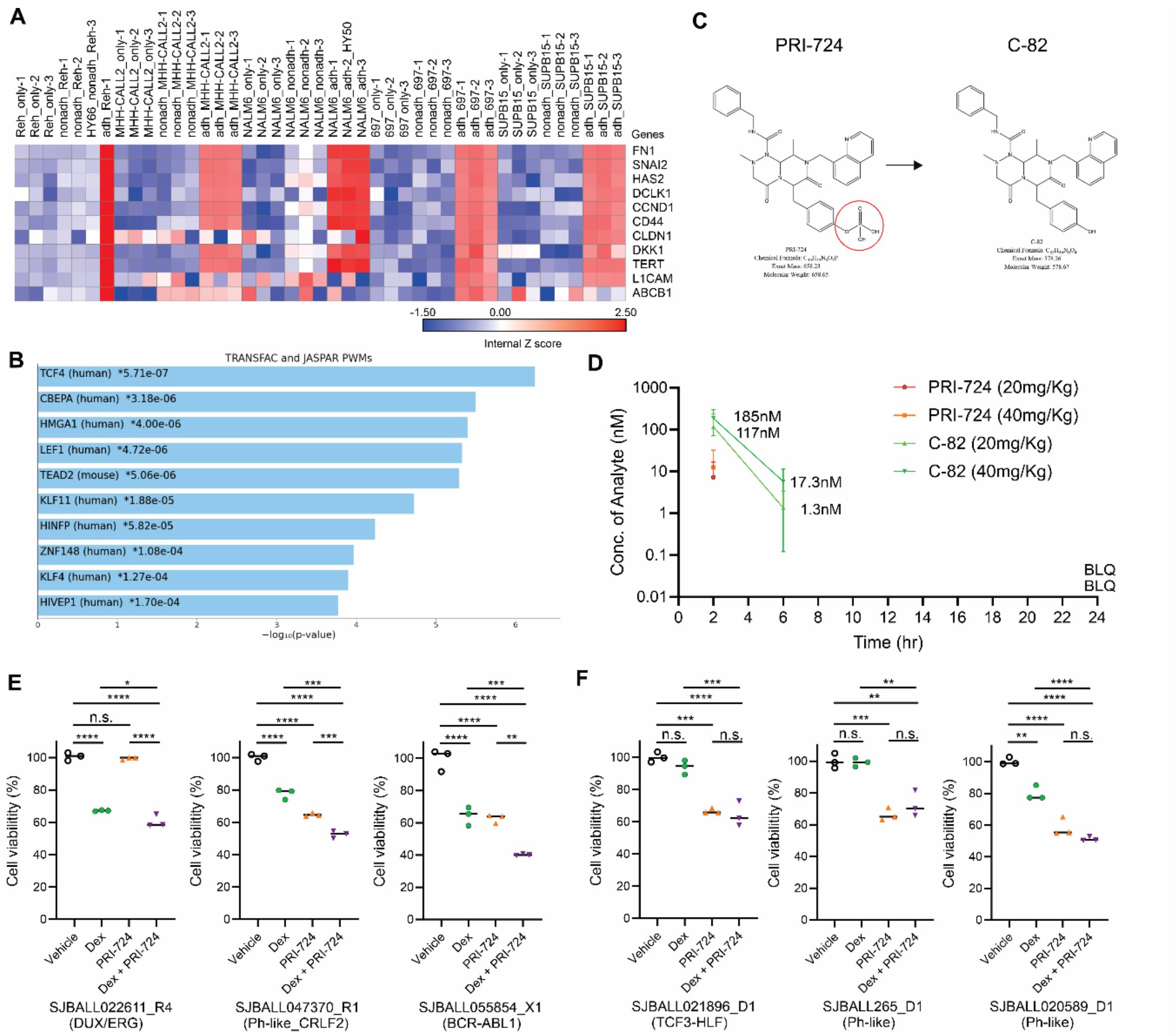

Figure S7. Enrichment of WNT/β-catenin target genes in Adh ALL cells; pharmacokinetic analysis of PRI-724; and effects of DEX, PRI-724, or the combination of PRI-724 and DEX in *ex vivo* B-ALL PDX, related to Figure 7. (**A**) Upregulation of WNT target genes associated with stemness and self-renewal in Adh ALL cells. Heatmap shows internal Z score. Genes from were analyzed.[60] (**B**) Genes retaining TCF4 and LEF1 binding site at the promoter were in Adh ALL cell lines. Bar chart visualization for TRANSFAC_and_JASPAR_PWMs by EnrichR. (**C**) PRI-724 is a pro-drug that is converted to its dephosphorylated form, C-82. (**D**) The concentration of PRI-724 and C-82 in the plasma at 2, 6, 24 hours post injection of PRI-724 (20 and 40 mg/kg) by intraperitoneal (IP) injections into NSG mice. The concentrations of drugs were measured by LC-MS; n=3 per group. The concentration of C-82 in the plasma of mice treated with either 20 or 40 mg/kg PRI-724 reached 117 nM and 185 nM, respectively, at 2 hours. However, it rapidly declined to 17.3 nM and 1.3 nM, respectively, at 6 hours. BLQ: below the lower limit of quantitation. Data, mean ± SD. (**E**) B-ALL PDX showing response to PRI-724 and additive effect with combination of PRI-724 and DEX (n=3). B-ALL PDX cells marked with luciferase and YFP (vCL20SF2-Luc2a-YFP) were used for cell viability assays. Data: individual value with mean. (**F**) B-ALL PDX showing response to PRI-724 but no additive effect on combination of PRI-724 and DEX (n=3). n.s.: not significant. Data, individual value with mean. Statistical testing was performed using one-way ANOVA with Tukey's multiple comparisons test, ****p <0.0001, ***p <0.0005, **p <0.005, *p < 0.05.
